# NAF-MEEF: A Nonlinear Activation-Free Network Based on Multi-Scale Edge Enhancement and Fusion for Railway Freight Car Image Denoising

**DOI:** 10.3390/s25092672

**Published:** 2025-04-23

**Authors:** Jiawei Chen, Jianhai Yue, Hang Zhou, Zhunqing Hu

**Affiliations:** 1 School of Mechanical, Electronic and Control Engineering, Beijing Jiaotong University, Beijing 100044, China; 21116024@bjtu.edu.cn (J.C.); zhqhu@bjtu.edu.cn (Z.H.); 2 School of Electronic and Information Engineering, Beijing Jiaotong University, Beijing 100044, China; hangzhou@bjtu.edu.cn

**Keywords:** image denoising, NAFNet, learnable Sobel convolution, attention mechanism, composite loss function

## Abstract

Railwayfreight cars operating in heavy-load and complex outdoor environments are frequently subject to adverse conditions such as haze, temperature fluctuations, and transmission interference, which significantly degrade the quality of the acquired images and introduce substantial noise. Furthermore, the structural complexity of freight cars, coupled with the small size, diversity, and complex structure of defect areas, poses serious challenges for image denoising. Specifically, it becomes extremely difficult to remove noise while simultaneously preserving fine-grained textures and edge details. These challenges distinguish railway freight car image denoising from conventional image restoration tasks, necessitating the design of specialized algorithms that can achieve both effective noise suppression and precise structural detail preservation. To address the challenges of incomplete denoising and poor preservation of details and edge information in railway freight car images, this paper proposes a novel image denoising algorithm named the Nonlinear Activation-Free Network based on Multi-Scale Edge Enhancement and Fusion (NAF-MEEF). The algorithm constructs a Multi-scale Edge Enhancement Initialization Layer to strengthen edge information at multiple scales. Additionally, it employs a Nonlinear Activation-Free feature extractor that effectively captures local and global image information. Leveraging the network’s multi-branch parallelism, a Multi-scale Rotation Fusion Attention Mechanism is developed to perform weight analysis on information across various scales and dimensions. To ensure consistency in image details and structure, this paper introduces a fusion loss function. The experimental results show that compared with recent advanced methods, the proposed algorithm has better noise suppression and edge preservation performance. The proposed method achieves significant denoising performance on railway freight car images affected by Gaussian, composite, and simulated real-world noise, with PSNR gains of 1.20 dB, 1.45 dB, and 0.69 dB, and SSIM improvements of 2.23%, 2.72%, and 1.08%, respectively. On public benchmarks, it attains average PSNRs of 30.34 dB (Set12) and 28.94 dB (BSD68), outperforming several state-of-the-art methods. In addition, this method also performs well in railway image dehazing tasks and demonstrates good generalization ability in denoising tests of remote sensing ship images, further proving its robustness and practical application value in diverse image restoration tasks.

## 1. Introduction

The timely detection of component defects makes a significant contribution to the safety and stability of railway freight car operation. In recent years, more and more research has been devoted to using machine vision to solve the problem of component defect recognition, and image quality is an important prerequisite for defect detection, which directly affects the accuracy of the entire system. Therefore, improving image quality is crucial.

Due to the outdoor deployment of railway freight car image acquisition systems, the captured images are frequently affected by a variety of environmental disturbances such as fog, haze, dust, temperature fluctuations, and unstable signal transmission. These factors introduce significant noise and degradation during the stages of image acquisition, transmission, and reception. Additionally, image capture often occurs in poorly lit areas beneath the car body and is further challenged by complex structural occlusions, making the visualization of critical component details even more difficult. These issues severely constrain the accuracy of image-based defect detection tasks.

Unlike traditional image-denoising scenarios, railway freight car images face unique challenges. Defects are usually small in size, diverse in form, and complex in structure, while useful textures and edge structures are densely distributed and highly susceptible to noise interference. As a result, image denoising in this context must not only achieve robust noise suppression but also ensure the accurate preservation of fine-grained edge and texture information, rendering the task significantly more complex and specialized than general-purpose denoising problems.

Researching efficient methods for denoising railway freight car operation images is a crucial aspect of interdisciplinary fields, offering both theoretical significance and substantial practical application value [1,2]. The widespread application of digital image technology has made image denoising a key research topic in interdisciplinary fields such as medical imaging, satellite remote sensing, and video surveillance.

With the advancement of digital image technology in recent years, image denoising, as the most fundamental and important downstream task, plays a crucial role in improving image quality and the accuracy of subsequent tasks. In practical application scenarios, image data are subject to various interferences of noise. Image denoising aims to restore potential noise-free image data from image data contaminated by noise. However, this is an ill-posed inverse process, and there is no unique solution [3].

Denoising methods can be divided into two categories: model-based and learning-based approaches. Due to the presence of similar or repetitive edge texture information in natural images, model-based methods can reduce artifacts caused by complex texture information during denoising by combining non-local self-similarity with sparse representation. So far, a large number of models based on this technology have been developed. The most representative technique is BM3D [4], which achieves image denoising by extracting self-similarity features in images and performing domain transformations on self-similar blocks. WNNM [5] applies the weighted nuclear norm minimization method to image denoising by leveraging the non-local self-similarity of images. Subsequently, many methods based on this type have been continuously proposed, such as MNL-tSVD [6], BM4D [7], slice-based dictionary learning [8], and so on. Although model-based methods have achieved notable results in the field of image denoising, their shortcomings are also quite apparent. Firstly, such techniques require the design of specific models for each individual denoising task. Secondly, there is a lack of universality among various data, and manual or semi-automatic parameter adjustments are required for the model. In addition, their convergence takes a long time. These challenges not only need to be addressed by such methods but also hinder, to some extent, the practical application of these technologies.

In recent years, deep learning methods have been widely applied in various fields of computer vision. Unlike model-based methods, learning-based methods aim to learn model parameters from data and obtain statistical information about images and noise through training the model and completing the mapping between noisy images and denoised images to achieve denoising. The learning of deep neural networks can be divided into two types: self-supervised learning and supervised learning. The self-supervised denoising methods represented by N2N [9], N2S [10], S2S [11], and VDN [12] lack flexibility in adjusting network parameters, and the extracted features cannot fully represent noise, making it difficult to obtain complex mapping relationships between noisy images and denoised images.

Supervised learning can effectively address the issues present in the aforementioned self-supervised denoising techniques. DnCNN [13] accelerates the training process and enhances model denoising performance by employing residual learning and batch normalization techniques. FFDNet [14] effectively solves the problem of blind denoising by using noisy image blocks and noisy mapping blocks as inputs to the network. ADNet [15] introduces a denoising convolutional neural network guided by an attention mechanism, enabling finer extraction of noise information from complex backgrounds, thus achieving superior denoising results. ADL [3] introduces an adversarial distortion learning-based denoising method, where both the denoiser and discriminator are implemented using an autoencoder architecture known as Efficient-UNet. This approach effectively mitigates overfitting during training and improves the model’s denoising performance. DRAN [16] removes noise from images by integrating attention mechanisms and dynamic convolution operations while preserving critical image details. This network design utilizes the correlation between features and optimizes the propagation of residual features through spatial gating mechanisms, thereby improving denoising performance.

On this basis, recent research has further explored denoising mechanisms in specific task scenarios. Yang et al. propose DIPKD [17], which enhances lightweight SAR object detection via Selective Noise Suppression, Knowledge Level Decoupling, and Reverse Information Transfer, effectively filtering speckle noise and boosting student model performance. Saidulu and Muduli [18] designed DP-LDCTNet for low-dose CT denoising, combining Dynamic Convolution, a Structure-aware Network trained with contrastive learning, and CT-specific perceptual loss to preserve structural integrity. For low-light enhancement, Wang and Yuan [19] propose FIHN, integrating a hierarchical structure (TRGF and DDCF modules) with an invertible flow network trained using negative log-likelihood loss, improving contrast, noise suppression, and detail preservation. Hein et al. [20] proposed PFCM (Poisson Flow Consistency Models), which extends the applicability of supervised diffusion models to medical imaging.

In the process of railway image acquisition, noise weakens the ability to represent image details due to various external factors, which significantly limits the accuracy of fault detection. Although deep neural networks have made significant progress in denoising natural and medical images and effectively improved image quality, their adaptability in the specific field of railway images is still insufficient, resulting in limited generalization ability. In addition, due to the scarcity of data in railway freight car image scenes, there are relatively few related studies and a lack of specialized optimized denoising algorithms. In practical applications, existing denoising methods often struggle to effectively preserve high-frequency textures and structural edges, which are crucial for downstream tasks such as defect localization and classification in railway freight car images. This deficiency can lead to problems such as blurred image contours and loss of key visual clues. Especially in complex railway freight car image backgrounds, accurately preserving the edge information of complex components and distinguishing noise from useful visual information remains a major challenge. These limitations result in poor performance of images in terms of content texture, detail restoration, and other aspects. Therefore, it is urgent to design denoising algorithms specifically tailored to the operating environment and visual complexity of railway freight car images in order to improve the reliability and accuracy of fault detection.

In response to the challenge of simultaneously improving noise suppression and edge information preservation in railway freight car images, this paper proposes a method called Nonlinear Activation-Free Network based on Multi-Scale Edge Enhancement and Fusion (NAF-MEEF), which leverages supervised learning concepts within Nonlinear Activation-Free Networks. The proposed algorithm adopts a fully convolutional architecture and is capable of effectively denoising railway freight car images.

To comprehensively validate its effectiveness, NAF-MEEF has been evaluated on both self-constructed railway freight car datasets and public datasets such as Set12 and BSD68 [21]. Additionally, denoising experiments on a remote sensing ship dataset and image dehazing experiments on railway freight car images were conducted, further demonstrating the robustness and generalization capability of the proposed method. The experimental results confirm that NAF-MEEF not only excels in railway freight car image denoising but also achieves competitive performance on diverse benchmark datasets. In summary, the main contributions of this article are as follows:

(1) This paper proposes a Multi-scale Edge Enhancement Initialization Layer, designed based on learnable Sobel convolution, which adaptively extracts high-frequency edge features of images at multiple scales.
(2) Dual-brand Nonlinear Activation-Free Network (D-NAFNet) is constructed as the core feature extractor of the algorithm. It adopts an efficient, lightweight UNet architecture and employs the Nonlinear Activation-Free Network Block (NAF-MEEF’s block) as its backbone, enabling hierarchical coordination during feature extraction.
(3) A Multi-scale Rotation Fusion Attention Mechanism is proposed that effectively integrates multi-scale information and establishes the relationship between channel and spatial attention.
(4) A composite loss function is introduced for the training phase that combines L1 loss with pyramidal textural loss, thereby preserving texture information in complex regions and minimizing noise amplification in non-textured areas.

The rest of this paper is organized as follows: Section 2 provides a detailed description of the proposed NAF-MEEF algorithm. Section 3 introduces the composite loss function used in this paper. Section 4 presents the experimental settings. Section 5 shows extensive experimental results and analysis. Section 6 conducts ablation studies. Section 7 concludes the paper.

## 2. Methods

**Figure 1:**
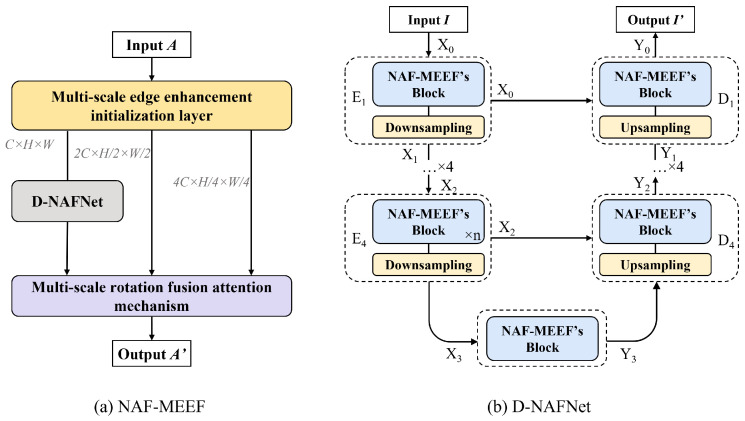
Denoising network framework. (**a**) The NAF-MEEF denoising network framework consists of a multi-scale initialized edge enhancement layer, a feature extractor D-NAFNet, and a Multi-scale Rotation Fusion Attention Mechanism; (**b**) The feature extractor D-NAFNet consists of the UNet framework and NAF-MEEF’s block.

NAF-MEEF (Figure 1a) aims to learn the mapping relationship between noisy images and clean images in order to effectively remove noise while preserving image details as much as possible. To achieve this goal, NAF-MEEF has enhanced its feature extraction and edge information preservation capabilities through multiple architectural innovations. Firstly, we designed a 2D dual-branch deep convolutional block (2DDCB) in the NAF-MEEF’s block and constructed a D-NAFNet feature extraction network using this block. Unlike directly using NAFNet as the backbone, we redesign the feature extraction pathway, incorporating D-NAFNet (Figure 1b) as an integral part of the overall architecture. In addition, the proposed Multi-scale Edge Enhancement Initialization Layer (MEEIL) integrates learnable Sobel operators across multiple scales to enhance edge representation at the early stage of image input. Furthermore, a Multi-scale Rotation Fusion Attention Mechanism is employed to adaptively fuse multi-scale features. These designs are not merely a simple stacking of modules but rather an organically integrated solution aimed at addressing common issues in images, such as edge degradation and structural blurring.

### 2.1. Multi-Scale Edge Enhancement Initialization Layer

This section introduces a Multi-scale Edge Enhancement Initialization Layer designed to address a critical limitation in image-denoising tasks—namely, the loss of fine edge and high-frequency details caused by strong or composite noise. Traditional convolutional layers often struggle to preserve such features, especially when processing images at varying resolutions or under severe noise corruption. To mitigate this, we propose a dedicated initialization layer that enhances edge-related features from the input image across multiple scales, thereby improving the model’s sensitivity to structural and high-frequency components from the very beginning of the network.

Specifically, the proposed layer consists of a multi-scale initialization structure that splits the input image into three resolution branches and applies a trainable Sobel operator at each scale [22]. Unlike conventional Sobel filters with fixed weights, our trainable Sobel operator learns to adaptively capture vertical, horizontal, and diagonal edge information by optimizing its learnable parameter α during training. This enables the model to generate edge-enhanced feature maps that are more robust to noise and better aligned with the underlying structural information of the image.

**Figure 2:**
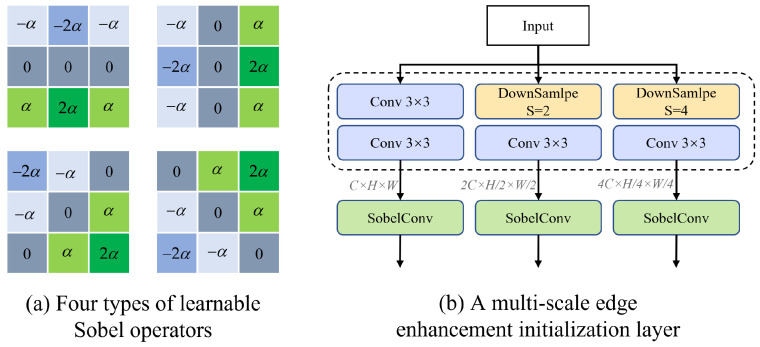
Illustration of (**a**) Learnable Sobel operator; (**b**) Multi-scale Edge Enhancement Initialization Layer (MEEIL).

This design is particularly motivated by the need for strong initialization in low-level vision tasks such as denoising, where edge preservation plays a vital role in visual quality. By introducing edge enhancement early in the network, the model is better guided during training to retain contours and fine textures, which are often degraded in standard convolutional pipelines. The structure of the trainable Sobel operator and the multi-scale fusion process is illustrated in Figure 2a.

(1) $$K_{1}=\left[\begin{array}{ccc} -\alpha & -2\alpha & -\alpha \\ 0 & 0 & 0\\ \alpha & 2\alpha & \alpha \end{array}\right],\;K_{2}=\left[\begin{array}{ccc} -\alpha & 0 & \alpha \\ -2\alpha & 0 & 2\alpha \\ -\alpha & 0 & \alpha \end{array}\right]\;K_{3}=\left[\begin{array}{ccc} -2\alpha & -\alpha & 0\\ -\alpha & 0 & \alpha \\ 0 & \alpha & 2\alpha \end{array}\right],\;K_{4}=\left[\begin{array}{ccc} 0 & \alpha & 2\alpha \\ -\alpha & 0 & \alpha \\ 2\alpha & -\alpha & 0 \end{array}\right]$$

As shown in Figure 2b, the input image *I* is first subjected to multi-scale processing to extract edge features at different scales. Specifically, three different scales of convolution branches are used, namely no downsampling, downsampling factor S=2, and downsampling factor S=4. The feature extraction process for each scale can be represented as:

(2) $$\begin{array}{c} I_{s}={\mathrm{Conv}}_{3\times 3}\left({\mathrm{Conv}}_{3\times 3}\left(\mathrm{Downsample}\left(I,s\right)\right)\right),s\in \left\{1,2,4\right\} \end{array}$$

After two 3×3 convolutions at each scale, the corresponding evidence graph $I_{s}$ obtained separately. After obtaining the evidence graph $I_{s}$ at different scales, we use Sobel convolution to extract the edge features at each scale, which can be expressed as follows:

(3) $$\begin{array}{c} O_{s}=SobelConv\left(I_{s}\right),s\in \left\{1,2,4\right\} \end{array}$$

This design ensures better preservation of edge features in the image, especially for denoising tasks, where enhanced high-frequency information is critical for effectively reducing noise while maintaining image details.

### 2.2. Feature Extraction Network: D-NAFNet

Convolutional neural networks (CNNs) have been widely adopted in computer vision tasks such as image denoising, object detection, and image segmentation, demonstrating significant effectiveness. With the rapid advancement of deep learning technologies, researchers have continuously optimized CNNs, yielding notable improvements in convolutional operation design [23,24,25,26] and overall network architecture refinement [27,28], all aimed at enhancing model performance and efficiency. Recently, the introduction of NAFNet [29] has offered a new perspective on image denoising by questioning the necessity of nonlinear activation functions in traditional CNNs. NAFNet proposes a model built from scratch that excludes these functions, showing they may not be essential for denoising tasks. This paradigm shift has inspired novel approaches focusing on edge preservation and multi-scale feature fusion to better capture fine-grained information. Complex denoising models often suffer from high computational costs due to their deep and parameter-heavy structures, which pose challenges in resource-constrained environments. In response, Chen et al. [29] proposed a model construction strategy that emphasizes structural simplicity by avoiding unnecessary components while iteratively refining key modules. Building on this idea, the method presented in this study aims to develop a streamlined, efficient feature extractor suitable for real-time denoising applications such as railway freight car image processing. Subsequent sections elaborate on architectural and block-level design choices guided by these principles.

#### 2.2.1. Architecture

**Figure 3:**
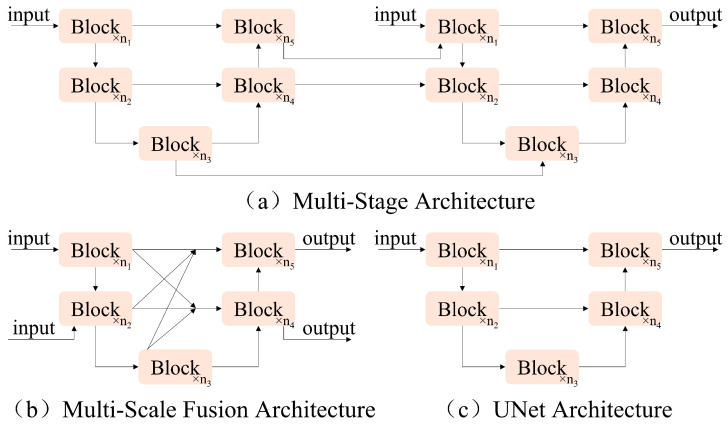
Comparison of different image restoration model architectures.

With the continuous development of deep learning, researchers continue to develop and innovate the model constructs; for example, some multi-stage architectures stack UNet networks in series (Figure 3a), and multi-scale fusion architectures enhance the fusion of features at different scales through complex inter-block connections (Figure 3b). In this paper, the classical single-stage UNet network architecture (Figure 3c) is adopted to ensure the simplicity of the model structure. Several state-of-the-art (SOTA) methods have used this single-stage UNet network so that the architecture does not become a hindrance to the model performance, as demonstrated by the subsequent experimental structure.

#### 2.2.2. PlainNet’s Block

**Figure 4:**
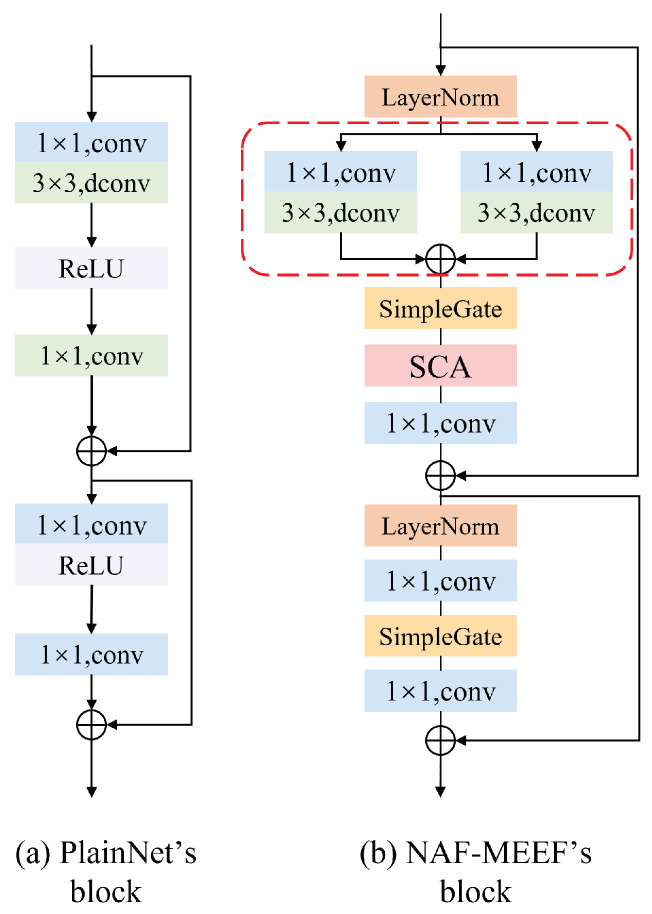
(**a**) The PlainNet’s block contains the most common components; (**b**) NAF-MEEF’s block is a novel nonlinear inactive network block proposed by us, which includes a dual-branch convolution block.

The main framework of deep neural networks is usually built through the stacking of modules, and the excellent design inside the modules largely determines the overall performance of the model. Therefore, this article will start with the most important and common components for combination, such as convolution operations, ReLU activation functions, and shortcuts. The combination of these basic components is shown in Figure 4a, which we call PlainNet’s block. In the design of the module, this article did not consider the introduction of Transformer structure, mainly based on the following two considerations: Firstly, several studies in recent years have shown that Transformer is not the only method that can achieve excellent denoising performance [29]. In addition, compared with self-attention mechanisms, convolutional neural networks have the advantages of simpler mechanisms and lower computational overhead and can achieve superior performance in situations where data volume is relatively limited.

The focus of this study is the denoising of railway freight car images; however, significant challenges exist in the acquisition and collection of such data samples. The available dataset is insufficient for training a Transformer model to a satisfactory level. Consequently, convolutional neural networks (CNNs) have been chosen as a cost-effective alternative, offering an optimal balance between performance and computational expense.

#### 2.2.3. NAF-MEEF’s Block

Normalization techniques are crucial in both upstream tasks (like image detection and segmentation) and downstream tasks (such as image denoising). Batch normalization [30] was initially introduced to address gradient vanishing and exploding issues during deep neural network training. However, it can become unstable with small batch sizes. To address this, instance normalization [31] was proposed, but its performance improvement is not always consistent across tasks. With the rise of Transformer-based methods [27,32,33,34], layer normalization [35] has gained widespread adoption, significantly boosting performance across various visual tasks. Thus, layer normalization techniques were incorporated into PlainNet to enhance model stability and generalization.

In our proposed NAF-MEEF architecture, layer normalization is applied at the beginning of each NAF-MEEF block and after the first residual connection (as shown in Figure 4b). This design helps stabilize feature distributions, reduce internal covariate shifts, and improve convergence performance under small-batch training conditions. Inspired by the successful application of LayerNorm in Transformer architectures, we incorporate it into our model to enhance stability and generalization across various types of noise and image domains.

Although the self-attention mechanism [36] has been widely applied in many tasks in recent years and has shown strong feature extraction capabilities, its complex structural design deviates from the original intention of simplifying the model in this paper. Therefore, this paper does not delve into its advantages and disadvantages. On the contrary, we improve model performance by introducing a simple channel attention mechanism [37]. This mechanism enhances overall performance by adaptively weighting data from different channels, allowing the model to focus more on important features within each channel.

**Figure 5:**
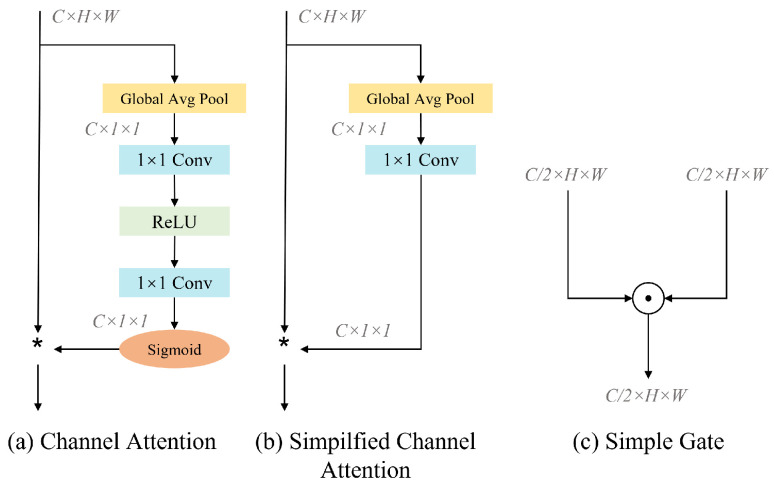
Illustration of (**a**) Channel Attention (CA), (**b**) Simplified Channel Attention (SCA), and (**c**) Simple Gate (SG). ⊙/∗: element-wise/channel-wise multiplication; C: channel.

Activation functions such as ReLU [38] and GELU [39] have been widely used in computer vision tasks and have achieved state-of-the-art results. But in this article, we borrowed the design of NAFNet [29] and introduced an activation method called Simple Gate, as shown in Figure 5c, which is a simple variant of GLU. This method divides the features into two equal parts in the channel dimension and introduces nonlinear components into the network through simple multiplication. This simplified activation function trims down the model structure while still maintaining performance, as expressed in the following equation:

(4) $$\begin{array}{c} \mathrm{simpleGate}(X,Y)=X⊙Y \end{array}$$

where *X* and *Y* are equally divided feature maps of equal size.

A common representation of the channel attention mechanism is shown in Figure 5a, where the feature maps are channel-weighted by spatial information squeezing as well as a multilayer perceptron. Where *X* represents the feature map, σ represents the sigmoid function, $W_{1}$ and $W_{2}$ represent the two fully connected layers, and max() represents the ReLU function between the fully connected layers.

(5) $$\begin{array}{c} CA\left(X\right)=X*\sigma (W_{2}\max(0,W_{1}\mathrm{pool}\left(X\right) \end{array}$$

The CA is simplified by retaining the global information aggregation capability of SimpleGate as well as the channel information interaction capability, as shown in the following equation and Figure 5b:

(6) $$\begin{array}{c} SCA\left(X\right)=X*Wpool\left(X\right) \end{array}$$

In addition, to improve the feature extraction ability of the model, we introduced a multi-path convolution structure in the convolution part of the model, as shown in the red dashed box in Figure 4b, called the 2D dual-branch deep convolution block (2DDCB) Specifically, the module consists of two parallel paths, with each path including a 1 × 1 pointwise convolution and a 3 × 3 depthwise convolution. These two paths independently process the input feature map and fuse their outputs via an element-wise addition operation. By combining pointwise convolution and depthwise convolution, the module captures fine-grained local features and learns global contextual information, thereby producing richer and more discriminative feature representations. This design ensures high computational efficiency while enhancing the ability to represent features effectively. It is highly suitable for extracting fine-grained and contextual features in tasks such as segmentation, detection, and denoising.

### 2.3. Multi-Scale Rotation Fusion Attention Mechanism

The human attention mechanism enables the selective processing of sensory input to guide behavior and decision-making. As an active and adaptive process, attention dynamically adjusts based on changes in external stimuli [40,41]. Computational models of attention have been widely adopted in computer vision and pattern recognition to predict attention allocation in visual and multimodal tasks. For instance, SENet [37] adaptively assigns weights to channels via learning, enhancing focus on informative features but failing to capture spatial information. CBAM [42] combines channel and spatial attention to more comprehensively capture image features, yet struggles to extract fine-grained and high-frequency details. Other approaches, such as A^2^-Nets [43], GSoP-Net [44], and GC-Net [45], introduce global dependency modeling via non-local operations, while modules like the Convolutional Triplet Attention Module [46], CCNet [47], and SPNet [48] improve contextual representation through cross-dimensional or intersecting attention structures.

Simultaneously, the extraction and integration of multi-scale features have become essential for enhancing feature representation in deep networks. Similar to the varying receptive fields in the visual cortex, deep models respond differently to inputs from multiple scales. HRNet [49] achieves multi-scale fusion through direct summation, but its rigid structure limits flexibility and discriminative power. Although CBAM [42] improves feature representation through dual attention, it overlooks the interplay between spatial and channel domains. These limitations highlight the need for a unified attention mechanism that can effectively fuse multi-scale features while preserving spatial-channel dependencies.

Motivated by the limitations of conventional attention mechanisms in effectively capturing fine-grained details across varied resolutions and inspired by the success of HRNet and MAFNet [50], this work proposes a novel attention mechanism that simultaneously integrates multi-scale features and models spatial–channel interactions. Traditional convolutional architectures often suffer from performance degradation when processing images at different resolutions, especially under noise corruption, due to insufficient integration of structural information across scales. To address this, we design an attention framework that not only preserves high-frequency details but also enhances context modeling by combining global and local interactions.

**Figure 6:**
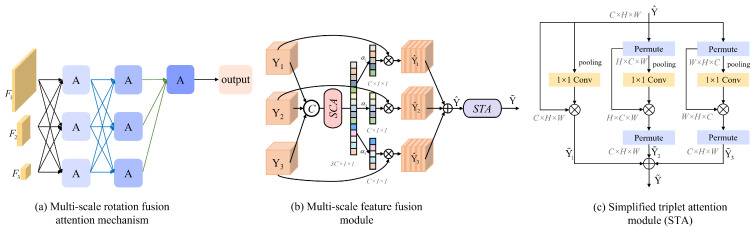
Illustration of (**a**) Multi-scale Rotation Fusion Attention Mechanism, where features of different scales are assigned weights and fused through the proposed attention mechanism; (**b**) multi-scale feature fusion module, where SCA assigns weights to features of different scales; (**c**) Simplified Convolutional Triple Attention Mechanism (STA).

The proposed method comprises two key components: a multi-scale feature fusion module and a simplified triplet attention (STA) module, as illustrated in Figure 6b,c. The multi-scale fusion module adaptively assigns weights to features from different scales using a simplified channel attention (SCA) mechanism, enabling effective cross-resolution interaction. Meanwhile, the STA module further refines the fused features by modeling dependencies across spatial and channel dimensions in a lightweight manner.

This design is grounded in the multi-scale edge enhancement initialization introduced earlier, which already improves low-level structural information extraction. Building upon this, the proposed module enhances mid- and high-level representation by fusing hierarchical feature maps. The adaptive weighting strategy ensures that essential details are preserved while reducing redundancy, ultimately improving the network’s denoising capacity in complex image-processing tasks.

To unify features from different resolution branches, upsampling and downsampling operations are applied, as shown in Figure 6a, aligning the features to the same scale for subsequent concatenation and fusion.

(7) $$Y=Concat(Y_{1},Y_{2},Y_{3})$$

where $Y_{1}$, $Y_{2}$, and $Y_{3}$ are feature maps obtained by a splicing operation with dimensions 3C×1×1.

*Y* then employs SCA to count the compact features of the channel along its spatial direction and eventually provides the corresponding feature descriptors for the three input features, each of which has a dimension of 3C×1×1, to obtain the attention weights $\alpha_{i}$.

The three generated attentional weights $\alpha_{i}$ will be used to multiply with the input features $Y_{i}$ to recalibrate the importance of the input information of the different multi-scale features as expressed in the following equation:

(8) $$\hat{Y_{i}}=Y_{i}⊙\alpha_{i}$$

The weighted feature maps $\hat{Y_{1}}$, $\hat{Y_{2}}$, and $\hat{Y_{3}}$ are summed and passed through the rotating attention machine module. As a subsequent operation of feature fusion, it consists of three parallel branches, two of which are responsible for capturing the cross-dimensional interactions between the channel dimension *C* and the spatial dimensions *H* and *W*. The last remaining branch is used to construct spatial attention similar to CBAM, as shown in Figure 6c.

This cross-dimensional interaction addresses the issue of missing dependency relationships between spatial and channel dimensions by capturing their mutual interactions. Each branch computes the descriptor similarly to the spatial-channel attention (SCA) mechanism, but the pooling method has been modified to incorporate *z*-pooling. Specifically, the pooling layer adjusts dimension 0 to dimension 2 by concatenating the average-pooled and maximum-pooled features along that axis. This method enhances the ability of layers to preserve rich feature representations.

As shown in Figure 6c, in the first branch, the input $\chi_{1}$ is rotated 90° anticlockwise along the H-axis, and the rotated tensor shape is W×H×C. The tensor shape is changed to 2×H×C by *z*-pool, and finally, a convolution is performed to generate a desired attention weight γ with the shape of 1×H×C, and subsequently, the attention weight γ is applied to the $\chi_{1}$, which is then rotated 90° clockwise along the *H*-axis, thus preserving the original input shape. An approximate operation is also taken in the second branch, the only difference being that the input $\chi_{2}$ is rotated and recovered along the *W*-axis. In the third branch, no rotation is required, and attention is constructed directly. The refined C×H×W tensor generated by the three branches is aggregated by the above operations.

$R_{i}\left(X\right)$ denotes the rotation operation, where i=1,2,3 represents the different rotation operations of the tensor, respectively, where $R_{1}$ represents no rotation; $Conv_{i}\left(X\right)$ represents the 1×1 convolution on path *i*; $Pool\left(X\right)$ denotes the pooling operation described above; and $R_{i}^{-1}$ denotes the inverse rotation operation in which the output features of path *i* are restored to their original dimensions.

The output of each path is as follows:

(9) $$\begin{array}{c} {\tilde{Y}}_{i}=R_{i}^{-1}\left(\mathrm{Pool}\left({\mathrm{Conv}}_{i}\left(R_{i}\left(\hat{Y}\right)\right)\right)\right),i\in \left(1,2,3\right) \end{array}$$

The final output $\tilde{Y}$ is an element-by-element summation of the three path outputs:

(10) $$\begin{array}{c} \tilde{Y}={\tilde{Y}}_{1}+{\tilde{Y}}_{2}+{\tilde{Y}}_{3} \end{array}$$

## 3. Loss Function

In the final stage of the denoising network, an attention mechanism was used to fuse multi-scale features in order to preserve the texture and details of the original image as much as possible and remove the noise in the image as much as possible. Further optimize the training of the network through a composite loss function, using L1 loss based on expressing the average absolute error between image pixels and the pyramid texture loss focused on preserving image details and texture features.

### 3.1. L1 Loss

In image denoising, loss functions such as L1, L2, and mean squared error (MSE) are widely used to improve Peak Signal-to-Noise Ratio (PSNR) and Structural Similarity (SSIM), thereby enhancing image quality. However, these traditional losses often fail to align with human visual perception, producing over-smoothed results lacking texture details [51]. To address this, perceptual metrics like SSIM [52], which considers brightness, contrast, and structure, and its extension MS-SSIM [53], which optimizes cross-scale structural similarity, have been proposed to preserve more details and reduce artifacts. Nevertheless, both methods still struggle to emphasize edges and textures.

Compared with L2 loss, which squares large errors and amplifies their impact, L1 loss computes absolute errors, making it more robust to outliers and better at preserving local structures. It typically yields more visually consistent reconstructions and retains fine details, particularly edge and texture features, thus preventing excessive blurring during denoising.

(11) $$\begin{array}{c} L_{L1}=E\{|F\left(y\right)-u\left|\right\} \end{array}$$

Among them, *y* is the noisy image, $F\left(y\right)$ is the denoised image after passing through the network (predicted value), *u* is the target image (actual value), $|F\left(y\right)-u|$ represents the degree of deviation between the predicted value and the actual value, and the absolute value is used to avoid the cancellation of positive and negative errors, thus reflecting the actual size of the error. Calculate the average absolute error using the expected *E*.

### 3.2. The Pyramid Texture Loss

The pyramid texture loss [54] aims to preserve the edges and textures of the image to be denoised without amplifying the side effects of noise in non-textured areas. Using a stationary wavelet transform called ‘algorithm à trous’ (ATW), this transform decomposes an image into several layers through a cubic spline filter and then subtracts any two consecutive layers to obtain a fine image with edges and textures. $\Delta_{j}$ represents the *j*-th layer texture image exported by ATW. *J* is the number of layers in the pyramid, representing the number of decomposition layers. Normally, four levels $\left(J=4\right)$ can already extract most of the edge and texture information. $\Delta_{j}\cdot F\left(y\right)$ represents the ATW transformation of the *j*-th layer on the $F\left(y\right)$ of the generated image. $\Delta_{j}\cdot u$ represents the ATW transformation of the J-th layer on the real image *u*. $|\Delta_{j}\cdot F\left(y\right)-\Delta_{j}\cdot u|$ represents the difference in the calculated image after the change.

(12) $$\begin{array}{c} L_{\mathrm{pyr}}=E\left\{\sum_{j=1}^{J}|\Delta_{j}\cdot F\left(y\right)-\Delta_{j}\cdot u|\right\} \end{array}$$

The pyramid texture loss effectively processes texture features of different sizes in image-denoising tasks through a multi-level approach. For small noise and large-scale structural changes, the pyramid texture loss can be effectively denoised through multi-level analysis, helping models recover small textures in images and avoid texture loss during denoising, especially in scenes with rich details and complex textures.

### 3.3. Composite Loss Function

In image denoising, it is important not only to remove noise but also to keep useful details like edges and textures. However, using only the L1 loss often leads to over-smoothed results, where fine details are lost.

To solve this problem, we introduce a composite loss that combines L1 loss with the pyramid texture loss [54]. The L1 loss helps reduce overall noise by minimizing pixel-level differences. The pyramid texture loss uses multi-scale wavelet decomposition to keep the texture and structural details at different levels. Together, they complement each other: L1 removes noise, while the texture loss preserves details.

This combination helps improve denoising quality, especially in images with rich textures. It also makes model training more stable and produces better-looking results. The final denoising loss function is obtained as shown in the formula:

(13) $$\begin{array}{c} L_{total}=\lambda_{L1}L_{L1}+\lambda_{P}L_{pyr} \end{array}$$

Among them, $\lambda_{L1}$ and $\lambda_{P}$ represent the weights of each loss, which can be based on experimental settings.

## 4. Experimental Setup

In this section, we present a comprehensive analysis of the influence of various design choices on the performance of the NAF-MEEF model introduced earlier. We then perform a series of experiments to evaluate the application of NAF-MEEF in restoring railway freight car images affected by different noise types, including Gaussian white noise, composite noise, and simulated real-world noise. Furthermore, the effectiveness of the proposed algorithm is validated on publicly available datasets to demonstrate its generalizability and broader applicability.

### 4.1. Dataset and Implementation Details

In this model, we implemented it using the PyTorch (version 1.12.0) framework. The computer configuration used for training includes an AMD 5600G CPU, 48 GB of RAM, and an NVIDIA RTX 3090 24 GB GPU. The initial weights of the network are set through random number initialization. Use AdamW algorithm for gradient update, with an initial learning rate of $10^{-3}$. The gradient descent strategy uses CosineAnnealingLR to adjust the learning rate, and the minimum learning rate at the end of training is $10^{-6}$. The self-built dataset and the public dataset use almost the same hyperparameter settings as described above, with the only difference being that the minimum learning rate at the end of training on the public dataset is $10^{-5}$.

Self-built dataset: To evaluate the denoising performance of NAF-MEEF on railway freight car images, a dataset comprising 3000 images of the sides and undersides of freight cars was constructed. Each image had a resolution of 512×512 pixels. The dataset was split into training and testing sets with an 8:2 ratio. Additionally, 80 images were set aside as a validation set to monitor the smoothness of the training process. The model uses railway freight car images with added Gaussian white noise, composite noise, simulated real-world noise, and haze as inputs, with clean images as targets for supervised training.

To verify the universality of the NAF-MEEF algorithm in image denoising and restoration tasks, over 4000 image data from the Waterloo Exploration Database [55] were used as the training set, including indoor, outdoor, natural scenery, and people, with high diversity. The scale of this dataset is moderate, which can meet the needs of most image processing algorithms without being too large, making it easy for experimental verification and performance comparison. All images are segmented into patches of 256×256 size for training denoising models.

To verify the robustness and generalization capability of the NAF-MEEF algorithm, we further conducted training and testing using 1341 remote sensing ship images from the MASATI-v2 [56] dataset, each with a resolution of 512×512 pixels. The dataset includes maritime scenes captured under various weather and lighting conditions. In the experiments, representative categories such as multiple ships and coastlines with ships were selected, offering rich semantic information that facilitates evaluating the model’s ability to preserve structural and texture details across different object categories. The dataset was divided into training, testing, and validation sets in a 9:3:1 ratio. The training set was further segmented into image patches of 256×256 pixels for training the denoising model.

Six sets of experiments were designed for preliminary data preparation to comprehensively evaluate the denoising ability of the proposed NAF-MEEF model:

(1) Gaussian White Noise Denoising Experiment on Railway Freight Car Images: Gaussian white noise with a mean of 0 and a standard deviation ranging from 0 to 55 is added to the training image to train a blind denoising model. Subsequently, Gaussian noise with standard deviations of 15, 25, and 50 was added separately for training non-blind denoising models.
(2) Composite noise denoising experiment on railway freight car images: Poisson noise, Gaussian noise with a mean of 0 and a standard deviation of σ∈[0,30], and salt and pepper noise with a noise density in the range of [0,30%] were added to the image to train a blind denoising model and evaluate its performance on the railway freight car image dataset.
(3) Simulation of real-world noise reduction experiment for railway freight car images: To accurately simulate the noise in the real world, a noise generator C2N [57] was introduced to synthesize real noise and train a denoising model, which was then evaluated on the railway freight car image dataset.
(4) Evaluation of NAF-MEEF performance on public datasets for blind image denoising: To validate the effectiveness and generalization ability of the NAF-MEEF algorithm, the publicly available dataset was used for training and evaluated on standard test sets Set12 and BSD68.
(5) Dehazing experiment of railway freight car images: To further verify the robustness of the model in practical railway application scenarios, a dehazing experiment of railway freight car images was constructed, using hazy images as input, training the model to restore clear images, and evaluating it on railway freight car datasets.
(6) Remote sensing ship image (MASATI-v2 [56]) denoising experiment: To verify the adaptability of the model in the remote sensing field, the MASATI-v2 dataset was selected for training and evaluation.

The proposed algorithm was evaluated against BM3D [3], WNNM [5], IRCNN [58], DnCNN [13], FFDNet [14], ADNet [15], MAFNet [54], and DRUNet [59] using railway freight car testing datasets. Two points merit attention: (1) BM3D and WNNM, as traditional algorithms, were excluded from comparisons involving composite and real-world noise due to their inherent limitations; (2) during the blind noise reduction comparison, noise level information was withheld from DURNet to ensure fairness in algorithm comparison. Additionally, comparative evaluations were conducted against algorithms such as CSF [60], TNRD [61], and ECNDNet [62] on the Set12 dataset to validate the effectiveness of the proposed algorithm using subjective perception and quantitative metrics.

### 4.2. Evaluation Criteria

The denoising effect is mainly compared from two aspects: visual subjective perception and quantitative indicators. Visual subjective perception can perceive the degree of subjective information retained in denoised images, and the denoising effect can be measured by qualitative analysis of the denoised images. Quantitative indicators measure the degree of deviation between the denoised image and the target image, with smaller deviations indicating superior denoising performance. Peak Signal-to-Noise Ratio (PSNR) and Structural Similarity Index (SSIM) are widely employed metrics for quantitative analysis and evaluation in image-denoising tasks.

PSNR is a widely used metric for assessing pixel-level differences between denoised images and target images, providing an indication of the model’s overall denoising performance [3,13,14,15,22]. A higher PSNR value signifies lower distortion and better quality of the denoised image. The PSNR metric is defined as follows:

(14) $$\begin{array}{c} \mathrm{PSNR}=10\mathrm{lg}[\frac{{\left(2^{n}-1\right)}^{2}}{\mathrm{MSE}}] \end{array}$$

MSE represents the root mean square error between the denoised image and the target image. PSNR ignores factors such as brightness and contrast when evaluating image quality, resulting in evaluation results that are inconsistent with subjective visual perception. Therefore, SSIM is introduced as another quantitative indicator.

SSIM can be calculated based on indicators such as luminance (L), contrast (C), and structure (S) by directly estimating the signal structure differences between the target image and the denoised image. SSIM can be represented as follows:

(15) $$\begin{array}{c} \mathrm{SSIM}\left(x,z\right)=\frac{\left(2\mu_{x}\mu_{z}+C_{1}\right)\left(2\sigma_{xz}+C_{2}\right)}{\left(\mu_{x}^{2}+\mu_{z}^{2}+C_{1}\right)\left(\sigma_{x}^{2}+\sigma_{z}^{2}+C_{2}\right)} \end{array}$$

Among them, $\mu_{x}$ and $\mu_{z}$ represent the mean values of image *x* and image *z*, respectively, while $\sigma_{x}^{2}$, $\sigma_{z}^{2}$, and $\sigma_{xz}$ represent the variance and covariance of image x and image z, respectively. $C_{1}$ and $C_{2}$ are constants, usually taken as $C_{1}={\left(k_{1}l\right)}^{2}$, $C_{2}={\left(k_{2}l\right)}^{2}$, $k_{1}=0.01$, $k_{2}=0.03$. The selection of these parameters follows the original definition of SSIM proposed by Wang et al. [52]. These values have been established as standard settings through extensive experimental validation on images with pixel values in the range of $\left[0,255\right]$, effectively preventing instability caused by near-zero means or variances in the denominator.

PSNR mainly focuses on the pixel values of the image and is sensitive to pixel distortion, while SSIM pays more attention to the structural information of the image. As can be seen from the formula, the smaller the root mean square error between the denoised image and the target image, the higher the PSNR, indicating that the image denoising effect is better. SSIM evaluates image structural similarity based on three aspects: brightness, contrast, and structure. The value ranges from 0 to 1, with higher values indicating greater similarity between images.

## 5. Experimental Results and Analysis

Before presenting the detailed results, we first summarize the scope of our experimental study. Specifically, our experiments were conducted on both real-world and publicly available datasets. For railway freight car images, we explored both denoising and defogging tasks to address the challenges posed by complex environments. To further evaluate the generalizability of the proposed method, additional denoising experiments were performed on the commonly used public datasets Set12 and BSD68, as well as on the remote sensing dataset MASATI-v2, which features maritime scenes captured under various weather conditions.

### 5.1. Gaussian White Noise Denoising Experiment on Railway Freight Car Images

**Figure 7:**
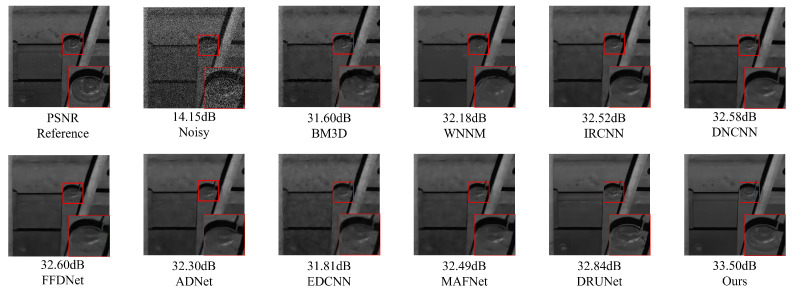
Comparison of local details in denoising railway freight car images using different algorithms.

In order to verify the Gaussian noise suppression effect of NAF-MEEF in railway freight car images, this paper first conducts Gaussian white noise denoising simulation experiments on railway freight car images. Peak Signal-to-Noise Ratio (PSNR) and Structural Similarity Index (SSIM) are employed as image quality evaluation metrics to assess the denoising performance of the algorithm. Figure 7 presents randomly selected, finely processed images from a railway freight car image dataset, illustrating the denoising effects of various methods under conditions of zero mean and a variance of 50. Figure 7 shows that other methods suffer from insufficient denoising or texture loss. The proposed method not only ensures denoising performance but also preserves more detailed information, achieving better reconstruction of edge details such as the ‘bolt and cotter pin’ in the image. Traditional methods such as BM3D and WNNM, although achieving certain denoising performance, exhibit texture blurring at the ‘cotter pin’ area, resulting in unclear visual imaging. Compared with traditional methods, ADNet and MAFNet can restore more image details. Partial texture details can be seen in the local detail map, but the reconstructed detail information is relatively messy and cannot effectively represent the image texture. The denoising performance of IRCNN, DNCNN, and FFDNet has been improved to some extent, but there is still blurring in some areas with rich structural information; DRUNet, as a suboptimal result, has a good denoising effect, but there is still some texture differences after zooming in on details. Compared with other methods, NAF-MEEF achieves fine filtering while preserving more edge detail information, resulting in optimal denoising and image restoration performance.

Table 1 and Table 2 present the average PSNR and SSIM parameter indicators achieved by various methods on the railway freight car dataset across five distinct noise levels: Level = 15, 25, 35, 45, and 50. The optimal results are highlighted in bold text in the tables. As shown in the tables, compared with the baseline methods, the proposed NAF-MEEF achieves an average PSNR gain of 2.45 dB, 1.84 dB, 1.40 dB, 1.22 dB, 1.24 dB, 1.71 dB, 2.4 dB, 1.44 dB, and 0.86 dB in removing Gaussian noise at Level = 50. NAF-MEEF not only yields the highest PSNR values but also the best SSIM scores. Overall, the method proposed in this paper outperforms other algorithms in terms of quantitative evaluation metrics across all five noise levels on the railway freight car image test set.

**Figure 8:**
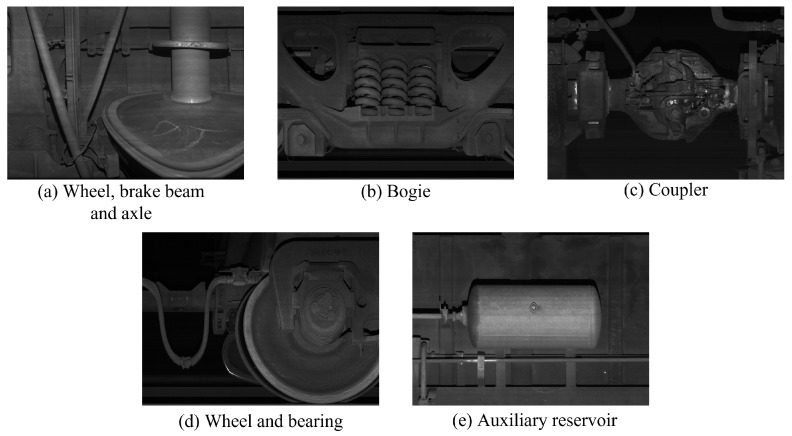
Railway freight cars are divided into five categories of large-sized images (1400×1024) based on actual operating scenarios.

To effectively evaluate the algorithm’s denoising performance on various components of railway freight cars, we divided the collected large-sized images (1400 × 1024) into five categories based on actual operational scenarios (as shown in Figure 8): (a) wheel, brake beam, and axle; (b) bogie; (c) coupler; (d) wheel and bearings; and (e) auxiliary reservoir. Gaussian white noise with a mean of 0 and variances of 15, 25, 35, 45, and 50 were added to railway freight images representing different locations, and the denoised PSNR and SSIM values were computed as metrics.

**Figure 9:**
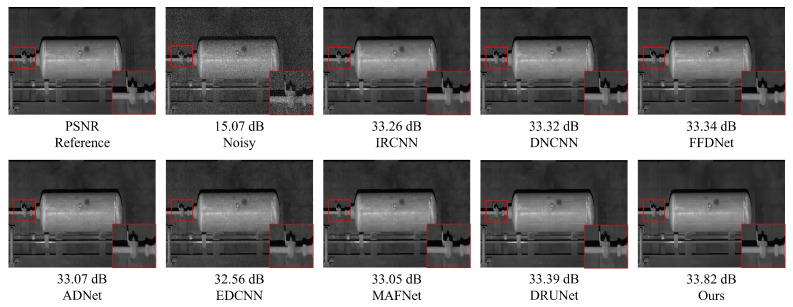
Comparison of local details of denoising effect in the auxiliary reservoir.

Figure 9 shows the denoising effect of NAF-MEEF and comparative algorithms on key component images of 1400 × 1024 large-sized railway freight cars (Level = 45). Although EDCNN can effectively remove noise from the images, it produces problems such as smooth details and information loss during the denoising process, which leads to blurry images after denoising. Due to the use of fixed filtering windows to extract features in DnCNN, FFDNet, and ADNet convolutions, it is impossible to supplement the information structure, resulting in the loss of some high-frequency information. Although MAFNet can preserve image details to a large extent, its use of cross-layer connection fusion features can lead to blurring of enlarged areas in the image. DRUNet can maintain relatively complete subjective information but is prone to producing some high-frequency artifacts. Compared with the above model, the proposed algorithm comprehensively utilizes the fusion of multi-scale features and efficient attention and constructs a composite loss function that can improve the denoising effect, content integrity, and subjective visual effect. It can effectively remove image noise while preserving image detail information as much as possible.

**Figure 10:**
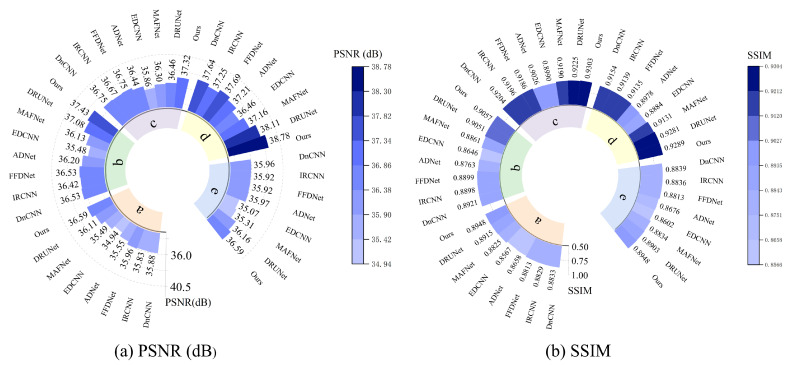
Illustration of denoising performance at different noise levels: (a–e) correspond to Gaussian noise levels of 15, 25, 35, 45, and 50, respectively. (**a**) shows the mean PSNR (dB) of denoising algorithms at each level; (**b**) presents the mean SSIM at each noise level. The best and second-best results are highlighted in bold and underlined, respectively. The color deepens as the value increases.

Figure 10 presents the comparative results of PSNR and SSIM metrics for different algorithms on the refined dataset comprising images of five key parts of railway freight cars. The tables indicate that the algorithm proposed in this study achieves higher PSNR and SSIM values than other methods across all five image types. Specifically, the proposed algorithm achieved average improvements in PSNR metrics of 0.48 dB, 0.35 dB, 0.57 dB, 0.47 dB, and 0.43 dB compared with the second-best results. Similarly, improvements in SSIM metrics were 0.0033, 0.0006, 0.0078, 0.0008, and 0.0045, respectively. The proposed method demonstrates the best denoising performance for different regions and parts of railway freight car images while also effectively preserving edge details. In summary, the algorithm achieves superior denoising results in terms of both subjective visual perception and objective image restoration fidelity.

### 5.2. Composite Noise Denoising Experiment on Railway Freight Car Images

**Figure 11:**
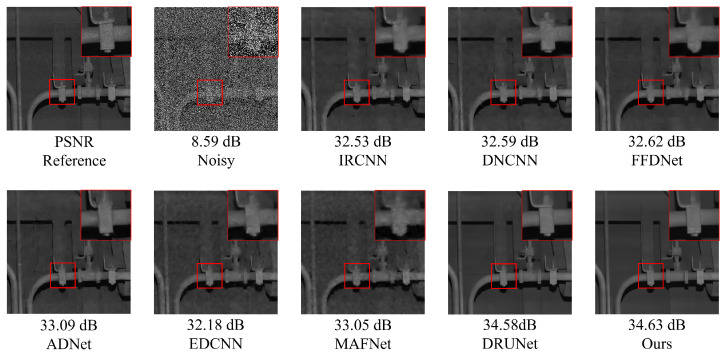
Comparison of denoising effects of different algorithms on Region 1 under the influence of composite noise, including Poisson noise, Gaussian noise (σ=30), and salt-and-pepper noise (level = 30).

**Figure 12:**
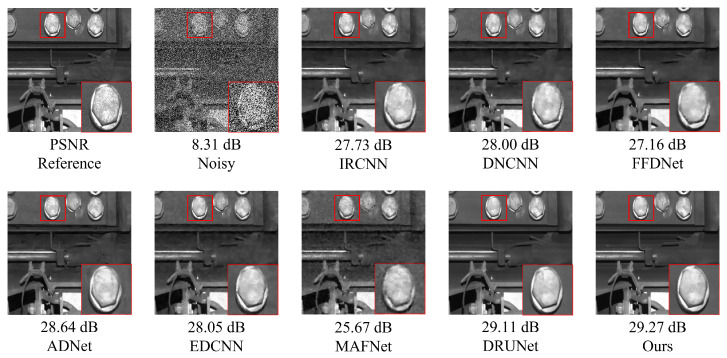
Comparison of denoising effects of different algorithms on Region 2 under the influence of composite noise, including Poisson noise, Gaussian noise (σ=30), and salt-and-pepper noise (level = 30).

Figure 11 and Figure 12 present examples of denoising results under the simultaneous influence of Poisson noise, Gaussian noise (σ = 30), and salt-and-pepper noise (level = 30) using various methods. As shown in the figure, the denoising performance of FFDNet and MAFNet is relatively poor. These methods not only fail to effectively remove noise from the image but also introduce significant blurriness, resulting in a substantial loss of image information. Although DnCNN, IRCNN, and EDCNN perform better in denoising, they are still unable to effectively mitigate detail artifacts generated during the process and fail to preserve the original image information adequately. Image distortion remains a significant issue. In contrast, ADNet and DRUNet show notable improvements in denoising performance and detail preservation; however, issues such as unclear edges persist. NAF-MEEF, on the other hand, provides excellent denoising and detail retention, resulting in a cleaner denoised image with clearer texture and richer high-frequency details in the enlarged areas, better aligning with visual expectations.

**Figure 13:**
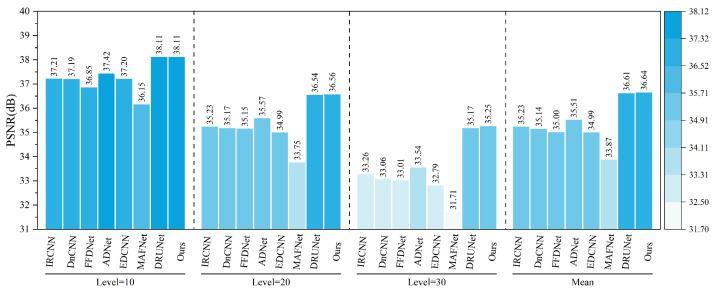
PSNR (dB) values for different methods applied to the railway freight car image datasets with composite noise levels of 10, 20, and 30. The color deepens as the value increases.

**Figure 14:**
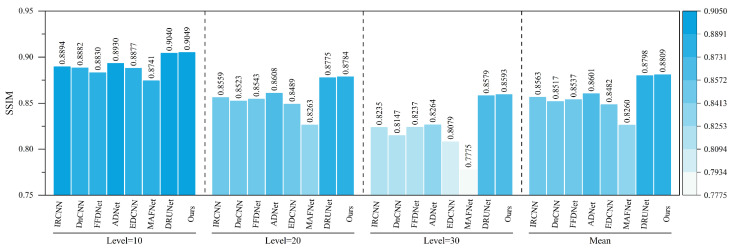
SSIM values for different methods applied to the railway freight car image datasets with composite noise levels of 10, 20, and 30. The color deepens as the value increases.

The quantitative indicators for removing composite noise from railway freight car images using different methods are shown in Figure 13 and Figure 14, where the level represents the sigma value of Gaussian white noise and the density level of salt and pepper noise. From the figures, it can be seen that the average PSNR of MAFNet is 2.77 lower than the proposed algorithm, which can be attributed to the fact that although its network adopts a multi-scale training method, it does not effectively fuse feature information of different scales. The average PSNR of IRCNN, DNCNN, FFDNet, and ADNet achieved better results compared with MAFNet but were 1.41 dB, 1.50 dB, 1.64 dB, and 1.13 dB lower than the NAF-MEEF algorithm, respectively. The average SSIM was 0.0246, 0.0292, 0.0272, and 0.0208 lower, which may be due to their relatively simple structure and shallow network layers, making it difficult to achieve high performance. There is still a lot of room for improvement in denoising quality in this complex, high-noise environment. DRUNet benefits from its combination of UNet and ResNet advantages, which can handle more complex noise. However, the PSNR and SSIM values of the method proposed in this paper are improved by 0.08 dB and 0.0014 in high-noise environments (level = 30). In summary, the proposed algorithm has good denoising effects in both visual subjective perception and image denoising and restoration approximation degree.

### 5.3. Simulation of Real-World Noise Reduction Experiment for Railway Freight Car Images

To address the challenge of lacking paired noisy–clean images in railway freight car scenarios, we utilize the Clean-to-Noisy (C2N) framework [57] to simulate real-world noise. Instead of retraining the C2N model, we directly adopt the pre-trained weights released by the original authors, which are trained on real-world noisy datasets (e.g., SIDD and DND). C2N is a generative noise modeling approach that learns to synthesize realistic noise maps from clean images without requiring any paired supervision or handcrafted noise assumptions. It includes both signal-dependent and signal-independent noise components and models spatial correlations to more accurately reflect real-world noise characteristics.

By using the C2N-generated noisy images, we are able to construct pseudo-paired data and train our denoising model in a supervised manner. This strategy is particularly suitable for our domain, where acquiring well-aligned training pairs is impractical due to environmental constraints. Although we do not modify or retrain the C2N model in this work, its integration allows us to better simulate realistic noise conditions and validate the effectiveness of our proposed denoising framework.

**Figure 15:**
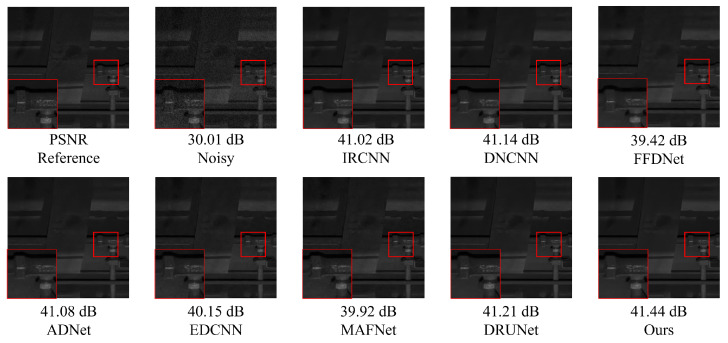
Under the influence of the generated real-world noise, different algorithms were applied to produce denoising results. For ease of comparison, the region of interest (ROI) marked within the red box was selected and magnified in the image.

Figure 15 shows the images selected from the test set that contain a large number of components. This type of image has complex textures and rich high-frequency information, which helps to reflect the denoising effect of different algorithms and their ability to preserve structural information. From the figure, it can be seen that MAFNet and FFDNet exhibit severe smearing of component positions in the denoised image and poor detail preservation in low-light conditions. In contrast, methods such as DnCNN, ADNet, DRUNet, and EDCNN perform better in detail restoration, but in locally enlarged images, the presentation of details still appears cluttered and fails to fully capture texture features. Compared with the above methods, the method proposed in this paper achieves a good denoising effect while more effectively preserving edge information.

Table 3 shows the average PSNR and SSIM parameter indicators taken by different methods on the test dataset under real-world noise, and the optimal values are highlighted in bold font. From the table, it can be seen that the proposed NAF-MEEF denoising algorithm has PSNR gains of 0.68 dB, 0.36 dB, 1.52 dB, 0.18 dB, 0.93 dB, 0.89 dB, and 0.27 dB compared with other comparative methods. At the same time, SSIM also achieved the best among all denoising methods.

### 5.4. Evaluation of NAF-MEEF Performance on Public Datasets for Blind Image Denoising

In this section, we train and test the performance of the NAF-MEEF method using publicly available datasets. It is important to emphasize that the core focus of this study is to design a denoising method specifically for railway freight car images. Since railway freight car images are grayscale images, experiments on the public datasets were also conducted exclusively on grayscale images. The experiments in this section aim to validate the performance and robustness of the proposed method in the context of publicly available datasets rather than pursuing state-of-the-art (SOTA) performance.

The main focus of this paper is the blind denoising task; therefore, non-blind denoising experiments were not conducted. The primary reason is that blind denoising is more aligned with real-world application scenarios: in practice, noise types are complex and difficult to predict accurately, whereas non-blind denoising methods rely on prior noise information, limiting their applicability in real-world scenarios. Hence, this study chooses the more challenging and practical blind denoising task to thoroughly evaluate the robustness and generality of the proposed method.

Although this study focuses on the blind denoising task, the results of the proposed method for blind denoising are compared with other methods on both blind and non-blind denoising tasks to comprehensively assess its performance and applicability. This comparison not only verifies the advantages of the proposed method in blind denoising but also demonstrates its potential and practical value in non-blind denoising tasks.

Table 4 and Table 5 present the PSNR metrics of NAF-MEEF on the Set12 and BSD68 datasets, respectively. (For the Set12 dataset, we ensured that the Lena image was not used during training and verified that its removal from the test set does not significantly affect the conclusions of this study.) As shown in Table 4, NAF-MEEF achieved the best performance on Set12 at noise levels of 15, 25, and 50. Compared with eight popular denoising methods listed in the table, the NAF-MEEF algorithm for blind denoising stands out, with PSNR improvements over DnCNN-B and ADNet-B of 0.41 dB, 0.40 dB, 0.52 dB, 0.32 dB, 0.31 dB, and 0.40 dB across various noise levels. Even compared with ADNet-S, which produces the second-best results for non-blind denoising, the blind denoising results of the proposed method achieved increases of 0.09 dB, 0.19 dB, and 0.36 dB at the three noise levels. At a noise level of 50, the proposed method demonstrated the most significant improvement, indicating its suitability for restoring highly noisy images. Table 5 further corroborates this finding. Although NAF-MEEF did not achieve the best performance at noise levels of 15 and 25, the differences in performance with mainstream algorithms were minor. When images were subjected to higher noise levels, the proposed method demonstrated superior image restoration capabilities, with PSNR improvements at a noise level of 50 of 0.78 dB, 0.53 dB, 0.43 dB, 0.17 dB, 0.17 dB, 0.21 dB, 0.17 dB, 0.11 dB, and 0.16 dB over other algorithms.

Overall, the NAF-MEEF method exhibits significant denoising advantages in high-noise environments. Its multi-scale edge enhancement initialization and Multi-scale Rotation Fusion Attention Mechanism effectively capture both local and global information of the image, enabling more accurate recovery of image details. This capability allows it to significantly outperform mainstream algorithms under high-noise conditions, further proving the potential applicability of the proposed method in real-world scenarios with complex noise.

### 5.5. Dehazing Experiment of Railway Freight Car Images

To further verify the robustness and generalization capability of the proposed method in practical railway application scenarios, a dehazing experiment based on railway freight car images was conducted, and performance was evaluated on a specifically constructed railway freight car image dataset. Haze synthesis was implemented using depth maps and the atmospheric scattering model. This approach utilizes the depth map to provide per-pixel distance information, where distant objects are more heavily obscured by haze while nearby objects remain relatively clear. By adjusting haze density, color, and transparency based on depth variation, the generated haze effects appear more realistic. Depth maps were produced using the Monodepth [63] method. To simulate different levels of degradation, three haze concentration levels—0.3, 0.6, and 0.9—were applied in the experiments.

**Figure 16:**
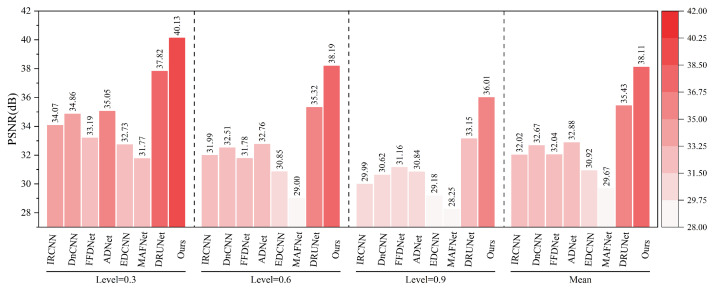
PSNR (dB) values of different methods applied to railway freight car image datasets with haze concentrations of 0.3, 0.6, and 0.9. The color deepens with increasing values.

**Figure 17:**
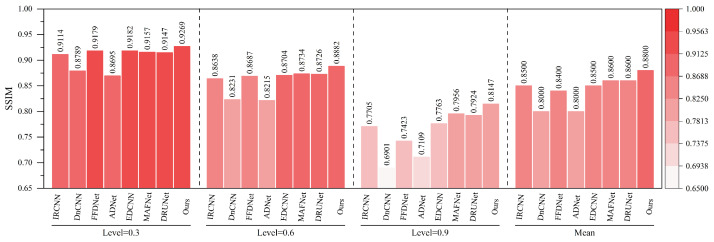
SSIM (dB) values of different methods applied to railway freight car image datasets with haze concentrations of 0.3, 0.6, and 0.9. The color deepens with increasing values.

As shown in Figure 16 and Figure 17, under various haze concentrations (0.3, 0.6, and 0.9), the proposed method demonstrates significant advantages in both PSNR and SSIM metrics.

In terms of PSNR, our method achieves an average value of 38.11 dB, which surpasses the second-best method, DRUNet (35.43 dB), by 2.68 dB. It also shows substantial improvements over traditional methods such as DnCNN (32.67 dB), IRCNN (32.02 dB), and FFDNet (32.04 dB). Notably, under the most severe haze condition (concentration 0.9), the proposed method still maintains a high PSNR of 36.01 dB, outperforming DRUNet (33.15 dB) by 2.86 dB, which highlights its robustness against heavy degradation. Regarding SSIM, our method achieves an average value of 0.9845, outperforming DRUNet (0.9780), ADNet (0.9520), and all other compared methods. Even under the most challenging haze conditions, the SSIM remains as high as 0.9751, indicating the model’s strong capability to preserve structural and textural information.

**Figure 18:**
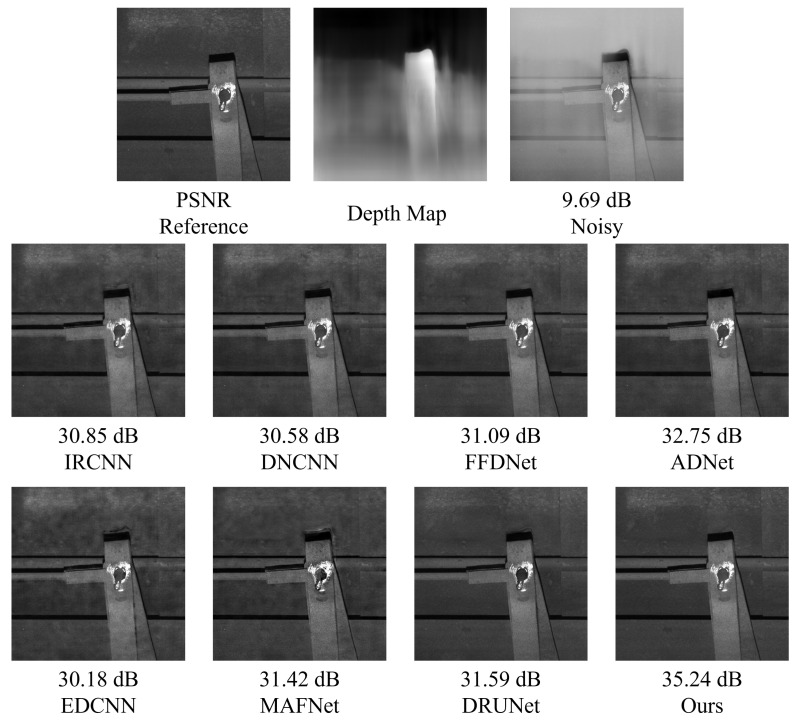
Denoising results of different algorithms under haze concentration level of 0.9.

As illustrated in Figure 18, different methods exhibit noticeable differences in the dehazing and denoising performance for railway freight car images. DnCNN, IRCNN, and FFDNet are limited in restoring structural and detailed information, often leading to over-smoothing or residual noise. Although DRUNet shows certain improvements in structure recovery, it still suffers from detail loss or minor artifacts. In contrast, the proposed method achieves the best performance in terms of structural preservation, edge sharpness, and highlight texture fidelity, fully demonstrating its adaptability and robustness in complex industrial imaging scenarios.

In summary, the proposed method exhibits superior performance in maintaining image fidelity and effectively handles various haze levels, verifying its potential for application in railway freight car image dehazing tasks.

### 5.6. Remote Sensing Ship Image Denoising Experiment

To further verify the generalization and robustness of the proposed method, additional denoising experiments were conducted on the publicly available remote sensing image dataset MASATI-v2. The experiments are divided into two parts: the first involves adding Gaussian noise with a standard deviation in the range of [0,55] to the images; the second introduces composite noise, which combines Poisson noise, Gaussian noise, and salt-and-pepper noise.

**Figure 19:**
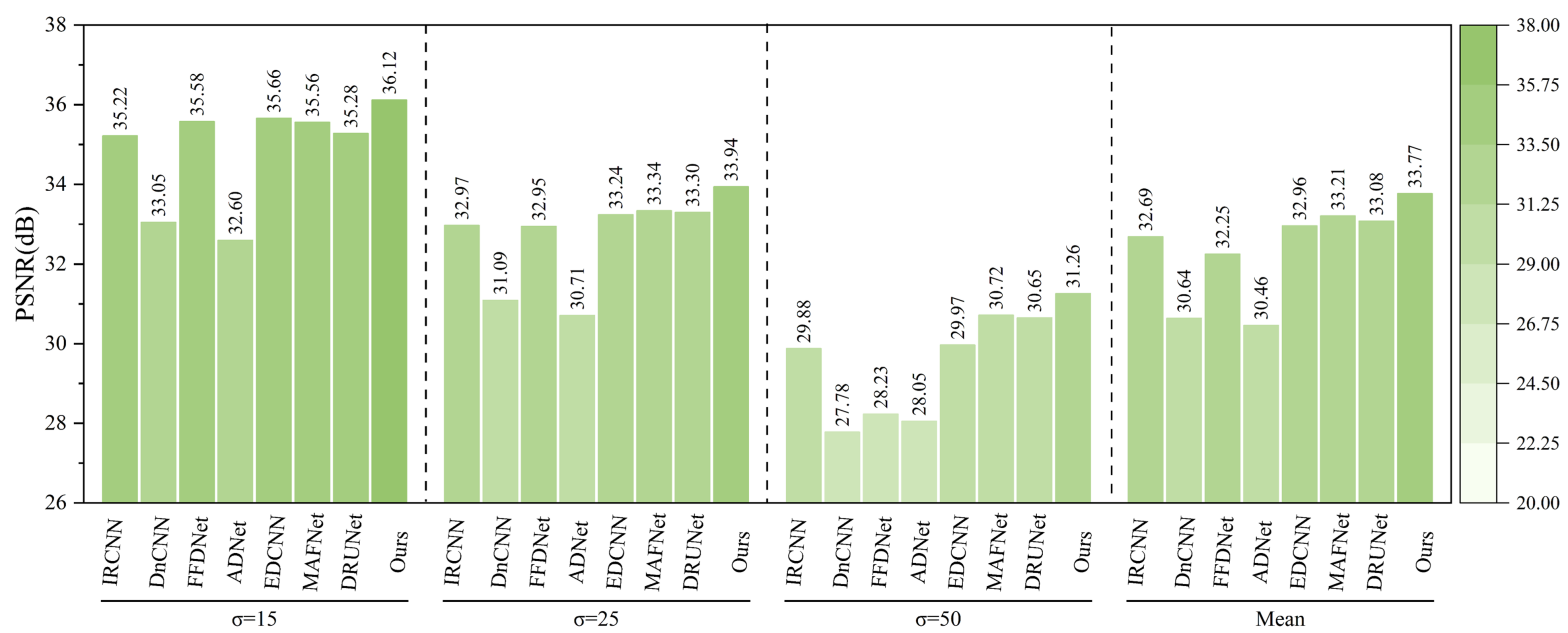
PSNR (dB) values of different methods applied to MASATI-v2 images under Gaussian noise with σ=15,25,50. The color deepens with increasing values.

As shown in Figure 19, after adding Gaussian noise with varying intensities (σ=15,25,50) to the MASATI-v2 remote sensing ship image dataset, the proposed method consistently outperforms existing denoising algorithms across all noise levels.

At the low noise level (σ=15), the proposed method achieves the highest PSNR of 36.12 dB, outperforming the second-best method, FFDNet (35.58 dB), and IRCNN (35.22 dB). As the noise level increases to σ=25 and σ=50, the proposed method maintains superior performance with PSNRs of 33.94 dB and 31.26 dB, respectively. Notably, under the highest noise level (σ=50), it still surpasses DRUNet (30.65 dB) and MAFNet (30.72 dB), demonstrating strong robustness.

**Figure 20:**
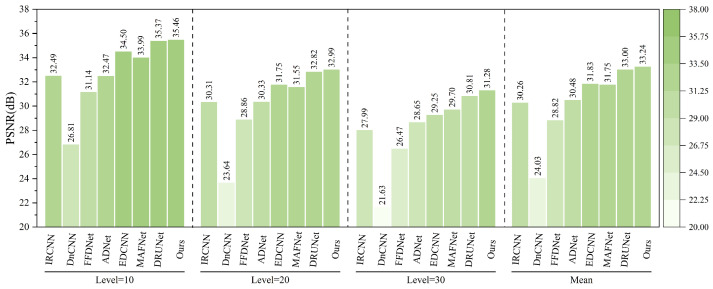
PSNR (dB) values of different methods applied to MASATI-v2 images under composite noise levels of 10, 20, and 30. The color deepens with increasing values.

As also presented in Figure 20, after introducing composite noise—comprising Poisson, Gaussian, and salt-and-pepper noise—into the MASATI-v2 dataset, the proposed method achieves the best PSNR results across all tested noise intensities (10, 20, 30), confirming its effectiveness in complex degradation scenarios. At a low noise level (10), our method reaches a PSNR of 35.46 dB, slightly outperforming DRUNet (35.37 dB) and significantly exceeding traditional methods such as DnCNN (26.81 dB) and FFDNet (31.14 dB). When the noise intensity increases to 20 and 30, the proposed method still maintains leading PSNR values of 32.99 dB and 31.28 dB, respectively. Overall, it achieves a mean PSNR of 33.24 dB across the three noise levels, surpassing DRUNet (33.00 dB), MAFNet (31.75 dB), and EDCNN (31.83 dB), highlighting its superior robustness and adaptability in multi-source noisy environments.

**Figure 21:**
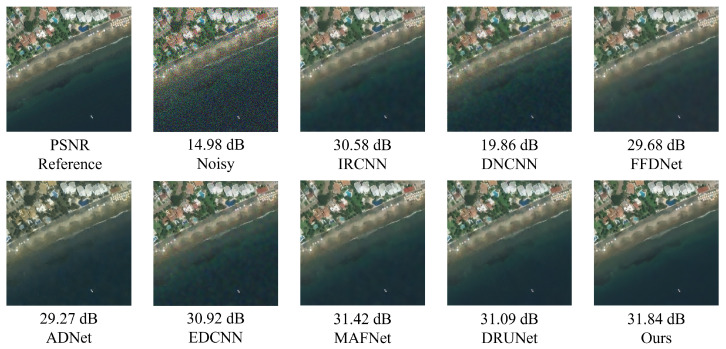
Visual comparison of MASATI-v2 images denoised by different algorithms under Gaussian noise (σ=50).

As shown in Figure 21, when Gaussian noise with a standard deviation of 50 is added, most compared methods exhibit over-smoothing or texture degradation, particularly around coastline edges and building structures. In contrast, the proposed method effectively removes the noise while preserving key semantic regions and structural details. The recovered coastlines appear sharp, building boundaries are well preserved, and the water surface is free from noticeable distortion, resulting in superior visual quality.

**Figure 22:**
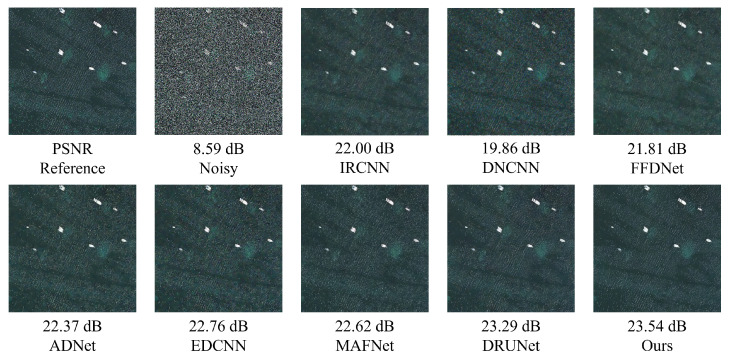
Visual comparison of denoising results on MASATI-v2 images under composite noise (level = 30) using different algorithms.

As shown in Figure 22, composite noise leads to severe degradation in visual quality, especially around the sea surface and small vessels, where heavy granular artifacts and structural blurring are observed. The denoising results of DnCNN and FFDNet remain suboptimal, with noticeable residual noise and blurred object boundaries. Although deep models such as DRUNet demonstrate improved edge preservation, they still suffer from texture degradation and slight artifacts. In contrast, the proposed method not only effectively suppresses various noise types but also preserves the original structural information of the image. The restored sea surface appears natural, and the contours of the ships are sharp and clear, demonstrating excellent visual fidelity.

In addition to achieving excellent performance in denoising railway freight car images, the proposed NAF-MEEF model also demonstrates strong denoising capability on the publicly available remote sensing image dataset MASATI-v2. This effectiveness is primarily attributed to the synergistic integration of the multi-scale initialization structure, the activation-free feature extraction architecture, and the Multi-scale Rotation Fusion Attention Mechanism. These components enable the NAF-MEEF network to learn noise-invariant feature representations. As a result, the proposed method consistently achieves leading denoising performance not only in specific industrial application scenarios but also in broader remote sensing benchmark evaluations, fully validating its robustness and generalizability.

## 6. Ablation Experiment

The ablation experiment is mainly aimed at denoising Gaussian white noise in railway freight car images. In order to verify the effectiveness of different modules in the proposed algorithm, the control variable method was used to measure the contribution of different modules to the model proposed in this paper. The experiment aims to remove multi-scale initialization edge enhancement layers and attention mechanisms and use a denoising network based on PlainNet’s block as the baseline network. Six ablation experiments were conducted under different module combinations with sigma values of 15, 25, 35, 45, and 50 added to the test images. The average PSNR and SSIM values under five different noise levels were used as quantitative indicators.

The setup of the ablation experiment is detailed in Table 6. Using PSNR as an example, the baseline PSNR value is 36.64 dB. Introducing layer normalization (LN) not only stabilizes the training process but also enhances the denoising effect, achieving a PSNR of 36.70 dB. SimpleGate (SG) and Simplified Channel Attention (SCA) integrate nonlinear capabilities and attention mechanisms into the network by replacing traditional activation functions and simplifying channel attention, leading to a 0.1 dB improvement in PSNR performance. 2DDCB, a minimalistic network structure, demonstrates that lightweight design can also yield gains for denoising tasks. Building on this foundation, the Multi-scale Edge Enhancement Initialization Layer (MEEIL) and Multi-scale Rotation Fusion Attention Mechanism (MRFAM) significantly enhance the network’s ability to preserve high-frequency information and facilitate smoother multi-scale information fusion. These cumulative enhancements improve the network’s performance, resulting in PSNR and SSIM increases of 0.23 dB and 0.0034, respectively, compared with the baseline.

The effect of the number of blocks on NAF-MEEF was verified in Table 7. The number of blocks was selected primarily based on the requirements of the feature extraction framework. When the number of blocks increased from 9 to 18, the PSNR value increased by 0.25 dB, the SSIM increased by 0.0012, and the model’s parameter count increased by 5.43 M. Despite the increase in parameters, the performance improvement supports this increase, with the model size remaining within a manageable range. When the number of blocks increased from 18 to 36, the performance improvement was marginal, while the parameter count increased substantially by 10.87M. Therefore, 18 blocks offer a balanced trade-off between performance and computational cost and were selected as the default option.

In addition, we conducted a comparative evaluation of the average inference time on 512 × 512 railway freight car images under the same GPU computing environment. As shown in Table 8, although the runtime of our method is slightly higher than that of lightweight models such as IRCNN (0.0029 s) or DnCNN (0.0067 s), it still meets the real-time requirements of practical industrial applications. In particular, the model variant using 18 basic blocks achieves a good balance between denoising performance and computational cost, with an average inference time of 0.0530 s. Considering its superior denoising capability and structural detail preservation, this model demonstrates strong practical applicability in real-world inspection systems, fulfilling the dual demands of image quality and processing efficiency.

## 7. Conclusions

To overcome the limitations of existing denoising algorithms in enhancing the denoising performance of railway freight car images while preserving edge details, this paper proposes a Nonlinear Activation-Free Network based on Multi-scale Edge Enhancement and Fusion (NAF-MEEF).The proposed algorithm enhances the ability to preserve high-frequency information at image edges through the MEEIL enhancement model, extracts features using D-NAFNet, and establishes an efficient multi-scale feature fusion mechanism using MRFAM. Finally, a composite loss function is constructed based on L1 loss and pyramid texture loss to iteratively adjust the network, thereby maximizing the removal of image noise. Compared with mainstream denoising algorithms, the experimental results validate the effectiveness of the proposed method. Objective evaluations show that the proposed algorithm achieves higher average PSNR and SSIM scores across multiple datasets, including the railway freight car image dataset, Set12, BSD68, and MASATI-v2. Subjective evaluations further indicate that the proposed method delivers better visual quality and detail preservation in most scenarios, demonstrating strong robustness and cross-domain adaptability. Future research will focus on evaluating the feasibility and performance of NAF-MEEF in single-image denoising tasks and further extending it to other image restoration applications such as image deblurring, compression artifact removal, and low-light image enhancement to verify its broader applicability and practical potential.

## Figures and Tables

**Table 1:** Denoising PSNR (dB) values of different algorithms at different noise levels. The best and second-best results are highlighted in bold and underlined, respectively.

Level	BM3D	WNNM	IRCNN	DnCNN	FFDNet	ADNet	EDCNN	MAFNet	DRUNet	Ours
15	37.25	37.20	37.53	37.61	37.58	37.47	36.96	37.17	37.87	**38.12**
25	35.58	35.71	36.10	36.21	36.18	36.01	35.43	35.85	36.42	**36.88**
35	34.32	34.51	35.08	35.22	35.20	34.91	34.28	34.93	35.44	**36.12**
45	33.27	33.83	34.24	34.40	34.38	33.96	33.29	34.16	34.72	**35.52**
50	32.80	33.41	33.85	34.03	34.01	33.54	32.85	33.81	34.39	**35.25**
Mean	34.64	34.93	35.36	35.49	35.47	35.18	34.56	35.18	35.77	**36.38**

**Table 2:** Denoising SSIM values of different algorithms at different noise levels. The best and second-best results are highlighted in bold and underlined, respectively.

Level	BM3D	WNNM	IRCNN	DnCNN	FFDNet	ADNet	EDCNN	MAFNet	DRUNet	Ours
15	0.8879	0.8930	0.8955	0.8973	0.8969	0.8946	0.8857	0.8942	0.9031	**0.9055**
25	0.8582	0.8688	0.8701	0.8721	0.8711	0.8659	0.8549	0.8690	0.8784	**0.8830**
35	0.8339	0.8479	0.8532	0.8555	0.8537	0.8428	0.8316	0.8534	0.8634	**0.8706**
45	0.8111	0.8393	0.8396	0.8419	0.8383	0.8198	0.8109	0.8414	0.8532	**0.8621**
50	0.8000	0.8315	0.8338	0.8357	0.8305	0.8091	0.8009	0.8360	0.8488	**0.8585**
Mean	0.8382	0.8561	0.8584	0.8605	0.8581	0.8464	0.8368	0.8588	0.8694	**0.8759**

**Table 3:** The PSNR (dB) and SSIM values of railway freight car datasets after denoising with different algorithms under the influence of generated real-world noise. The best and second-best results are highlighted in bold and underlined, respectively.

Method	DnCNN	IRCNN	FFDNet	ADNet	EDCNN	MAFNet	DRUNet	Ours
PSNR/dB	37.62	37.94	36.78	38.12	37.37	37.41	38.03	**38.30**
SSIM	0.9089	0.9152	0.8933	0.9204	0.9053	0.9113	0.9209	**0.9216**

**Table 4:** Average PSNR (dB) results of different methods on Set12 with noise levels of 15, 25, and 50. The best and second-best results are highlighted in bold and underlined, respectively.

Images	C.man	House	Pepper	Starfish	Monarch	Airplane	Parrot	Barbara	Boat	Man	Couple	Mean
Noise level		15										
BM3D	31.91	34.93	32.69	31.14	31.85	31.07	31.37	33.10	32.13	31.92	32.10	32.20
WNNM	32.17	35.13	32.99	31.82	32.71	31.39	31.62	**33.60**	32.27	32.11	32.17	32.54
TNRD	32.19	34.53	33.04	31.75	32.56	31.46	31.63	32.13	32.14	32.23	32.11	32.34
DnCNN	32.61	34.97	33.30	32.20	33.09	31.70	31.83	32.64	32.42	32.46	32.47	32.70
DnCNN-B	32.10	34.93	33.15	32.02	32.94	31.56	31.63	32.09	32.35	32.41	32.41	32.51
IRCNN	32.55	34.89	33.31	32.02	32.82	31.70	31.84	32.43	32.34	32.40	32.40	32.61
FFDNet	32.43	35.07	33.25	31.99	32.66	31.57	31.81	32.54	32.38	32.41	32.46	32.60
ECNDNet	32.56	34.97	33.25	32.17	33.11	31.70	31.82	32.41	32.37	32.39	32.39	32.65
ADNet	**32.81**	35.22	33.49	32.17	33.17	31.86	31.96	32.80	**32.57**	32.47	32.58	32.83
ADNet-B	31.98	35.12	33.34	32.01	33.01	31.63	31.74	32.55	32.48	32.34	32.43	32.60
Our-B	32.74	**35.61**	**33.51**	**32.45**	**33.29**	**31.90**	**32.03**	32.86	32.56	**32.53**	**32.67**	**32.92**
Noise level		25										
BM3D	29.45	32.85	30.16	28.56	29.25	28.42	28.93	30.71	29.90	29.61	29.71	29.78
WNNM	29.64	33.22	30.42	29.03	29.84	28.69	29.15	**31.24**	30.03	29.76	29.82	30.08
TNRD	29.72	32.53	30.57	29.02	29.85	28.88	29.18	29.41	29.91	29.87	29.71	29.88
DnCNN	30.18	33.06	30.87	29.41	30.28	29.13	29.43	30.00	30.21	30.10	30.12	30.25
DnCNN-B	29.94	33.05	30.84	29.34	30.25	29.09	29.35	29.69	30.20	30.09	30.10	30.18
IRCNN	30.08	33.06	30.88	29.27	30.09	29.12	29.47	29.92	30.17	30.04	30.08	30.20
FFDNet	30.10	33.28	30.93	29.32	30.08	29.04	29.44	30.01	30.25	30.11	30.20	30.25
ECNDNet	30.11	33.08	30.85	29.43	30.30	29.07	29.38	29.84	30.14	30.03	30.03	30.21
ADNet	**30.34**	33.41	31.14	29.41	30.39	29.17	29.49	30.25	30.37	30.08	30.24	30.39
ADNet-B	29.94	33.38	30.99	29.22	30.38	29.16	29.41	30.05	30.28	30.01	30.15	30.27
Ours-B	**30.34**	**33.81**	**31.18**	**29.92**	**30.56**	**29.28**	**29.64**	30.61	**30.48**	**30.19**	**30.43**	**30.58**
Noise level		50										
BM3D	26.13	29.69	26.68	25.04	25.82	25.10	25.90	27.22	26.78	26.81	26.46	26.51
WNNM	26.45	30.33	26.95	25.44	26.32	25.42	26.14	**27.79**	26.97	26.94	26.64	26.85
TNRD	26.62	29.48	27.10	25.42	26.31	25.59	26.16	25.70	26.94	26.98	26.50	26.62
DnCNN	27.03	30.00	27.32	25.70	26.78	25.87	26.48	26.22	27.20	27.24	26.90	26.98
DnCNN-B	27.03	30.02	27.39	25.72	26.83	25.89	26.48	26.38	27.23	27.23	26.91	27.01
IRCNN	26.88	29.96	27.33	25.57	26.61	25.89	26.55	26.24	27.17	27.17	26.88	26.93
FFDNet	27.05	30.37	27.54	25.75	26.81	25.89	26.57	26.45	27.33	27.29	27.08	27.10
ECNDNet	27.07	30.12	27.30	25.72	26.82	25.79	26.32	26.26	27.16	27.11	26.84	26.96
ADNet	27.31	30.59	27.69	25.70	26.90	25.88	26.56	26.64	27.35	27.17	27.07	27.17
ADNet-B	27.22	30.43	27.70	25.63	26.92	26.03	26.56	26.51	27.22	27.19	27.05	27.13
Ours-B	**27.45**	**31.26**	**27.88**	**26.47**	**27.17**	**26.08**	**26.67**	27.45	**27.60**	**27.37**	**27.42**	**27.53**

**Table 5:** Average PSNR (dB) of different methods on BSD68 with different noise levels of 15, 25, and 50. The best and second-best results are highlighted in bold and underlined, respectively.

Methods	BM3D	WNNM	TNRD	DnCNN	DnCNN-B	IRCNN	ECNDNet	ADNet	ADNet-B	Our-B
σ=15	31.07	31.37	31.42	31.72	31.61	31.63	31.71	**31.74**	31.56	31.27
σ=25	28.57	28.83	28.92	29.23	29.23	29.15	29.22	**29.25**	29.14	29.15
σ=50	25.62	25.87	25.97	26.23	26.23	26.19	26.23	26.29	26.24	**26.40**

**Table 6:** Experimental results for different module settings.

Baseline	LN	SG + SCA	2DDCB	MEEIL	MRFAM	PSNR	SSIM
✓						36.64	0.8810
✓	✓					36.70	0.8815
✓	✓	✓				36.80	0.8833
✓	✓	✓	✓			36.82	0.8838
✓	✓	✓	✓	✓		36.85	0.8843
✓	✓	✓	✓	✓	✓	36.87	0.8844

**Table 7:** The effect of the number of blocks.

	# of Block	PSNR	SSIM	Params(M)
	9	35.00	0.8573	6.48
NAF-MEEF	18	35.25	0.8585	11.91
	36	35.33	0.8597	22.78

**Table 8:** The average running time of different methods.

Methods	IRCNN	DnCNN	FFDNet	ADNet	EDCNN	MAFNet	DRUNet	Ours (#9)	Ours (#18)	Ours (#36)
Time (s)	0.0029	0.0067	0.0225	0.0065	0.0147	0.0279	0.0123	0.0363	0.0530	0.0877

## Data Availability

Data available on request due to restrictions (e.g., privacy, legal, or ethical reasons). The data presented in this study are available on request from the corresponding author due to confidentiality agreements. The dataset is based on real-world images of operating Chinese railway freight cars, which are subject to strict national confidentiality regulations. We regret that we are unable to publicly share or upload this dataset.

## References

[1] Jia X., Peng Y., Ge B., Li J., Liu S., Wang W. (2023). A multi-scale dilated residual convolution network for image denoising. Neural Process. Lett..

[2] Zhang W., Fan W., Yang X., Zhang Q., Zhou D. (2023). Lightweight single-image super-resolution via multi-scale feature fusion cnn and multiple attention block. Vis. Comput..

[3] Ghahremani M., Khateri M., Sierra A., Tohka J. (2024). Adversarial Distortion Learning for Medical Image Denoising. arXiv.

[4] Dabov K., Foi A., Katkovnik V., Egiazarian K. (2007). Image Denoising by Sparse 3-D Transform-Domain Collaborative Filtering. IEEE Trans. Image Process..

[5] Gu S., Zhang L., Zuo W., Feng X. Weighted nuclear norm minimization with application to image denoising. Proceedings of the IEEE Conference on Computer Vision and Pattern Recognition.

[6] Kong Z., Han L., Liu X., Yang X. (2018). A New 4-D Nonlocal Transform-Domain Filter for 3-D Magnetic Resonance Images Denoising. IEEE Trans. Med Imaging.

[7] Maggioni M., Katkovnik V., Egiazarian K., Foi A. (2013). Nonlocal Transform-Domain Filter for Volumetric Data Denoising and Reconstruction. IEEE Trans. Image Process..

[8] Papyan V., Romano Y., Sulam J., Elad M. Convolutional dictionary learning via local processing. Proceedings of the IEEE International Conference on Computer Vision.

[9] Lehtinen J., Munkberg J., Hasselgren J., Laine S., Karras T., Aittala M., Aila T. Noise2Noise: Learning Image Restoration without Clean Data. Proceedings of the 35th International Conference On Machine Learning.

[10] Batson J., Royer L. Noise2Self: Blind Denoising by Self-Supervision. Proceedings of the 36th International Conference on Machine Learning.

[11] Quan Y., Chen M., Pang T., Ji H. Self2Self with Dropout: Learning Self-Supervised Denoising from Single Image. Proceedings of the 2020 IEEE/CVF Conference on Computer Vision and Pattern Recognition (CVPR).

[12] Yue Z., Yong H., Zhao Q., Zhang L., Meng D. Variational Denoising Network: Toward Blind Noise Modeling and Removal. Proceedings of the Advances in Neural Information Processing Systems 32 (NIPS 2019).

[13] Zhang K., Zuo W., Chen Y., Meng D., Zhang L. (2017). Beyond a Gaussian Denoiser: Residual Learning of Deep CNN for Image Denoising. IEEE Trans. Image Process..

[14] Zhang K., Zuo W., Zhang L. (2018). FFDNet: Toward a Fast and Flexible Solution for CNN-Based Image Denoising. IEEE Trans. Image Process..

[15] Tian C., Xu Y., Li Z., Zuo W., Fei L., Liu H. (2020). Attention-guided CNN for image denoising. Neural Netw..

[16] Sharif S.M.A., Naqvi R.A., Biswas M. (2020). Learning Medical Image Denoising with Deep Dynamic Residual Attention Network. Mathematics.

[17] Yang X., Sun J., Duan S., Cheng D. Dual Information Purification for Lightweight SAR Object Detection. Proceedings of the AAAI Conference on Artificial Intelligence.

[18] Saidulu N., Muduli P.R. (2025). Dynamic Perception-oriented Low-dose CT Image Denoising Network using Structure-aware Self-similarity. Circuits Syst. Signal Process..

[19] Wang Y., Yuan X., Kang H., Chen Y., Li Y. (2025). Flow Learning-Based Image Hierarchical Structure Network for Low-Light Image Enhancement. SSRN.

[20] Hein D., Stevens G., Wang A., Wang G. (2025). PFCM: Poisson flow consistency models for low-dose CT image denoising. IEEE Trans. Med. Imaging.

[21] Roth S., Black M.J. (2009). Fields of experts. Int. J. Comput. Vis..

[22] Liang T., Jin Y., Li Y., Wang T. EDCNN: Edge enhancement-based Densely Connected Network with Compound Loss for Low-Dose CT Denoising. Proceedings of the 2020 15th IEEE International Conference on Signal Processing (ICSP).

[23] Chen Y., Dai X., Liu M., Chen D., Yuan L., Liu Z. Dynamic Convolution: Attention Over Convolution Kernels. Proceedings of the 2020 IEEE/CVF Conference on Computer Vision and Pattern Recognition (CVPR).

[24] Han K., Wang Y., Tian Q., Guo J., Xu C., Xu C. GhostNet: More Features From Cheap Operations. Proceedings of the 2020 IEEE/CVF Conference on Computer Vision and Pattern Recognition (CVPR).

[25] Frants V., Agaian S., Panetta K. (2023). QCNN-H: Single-Image Dehazing Using Quaternion Neural Networks. IEEE Trans. Cybern..

[26] Chollet F. Xception: Deep Learning with Depthwise Separable Convolutions. Proceedings of the 2017 IEEE Conference on Computer Vision and Pattern Recognition (CVPR).

[27] Liu Z., Mao H., Wu C.Y., Feichtenhofer C., Darrell T., Xie S. A ConvNet for the 2020s. Proceedings of the 2022 IEEE/CVF Conference on Computer Vision and Pattern Recognition (CVPR).

[28] Ronneberger O., Fischer P., Brox T. U-Net: Convolutional Networks for Biomedical Image Segmentation. Proceedings of the Medical Image Computing and Computer-Assisted Intervention—MICCAI 2015.

[29] Chen L., Chu X., Zhang X., Sun J. Simple Baselines for Image Restoration. Proceedings of the Computer Vision—ECCV 2022.

[30] Ioffe S., Szegedy C. Batch Normalization: Accelerating Deep Network Training by Reducing Internal Covariate Shift. Proceedings of the 32nd International Conference On Machine Learning.

[31] Ulyanov D., Vedaldi A., Lempitsky V. (2017). Instance Normalization: The Missing Ingredient for Fast Stylization. arXiv.

[32] Liu Z., Lin Y., Cao Y., Hu H., Wei Y., Zhang Z., Lin S., Guo B. Swin Transformer: Hierarchical Vision Transformer using Shifted Windows. Proceedings of the 2021 IEEE/CVF International Conference on Computer Vision (ICCV).

[33] Wang Z., Cun X., Bao J., Zhou W., Liu J., Li H. Uformer: A General U-Shaped Transformer for Image Restoration. Proceedings of the 2022 IEEE/CVF Conference on Computer Vision and Pattern Recognition (CVPR).

[34] Zamir S.W., Arora A., Khan S., Hayat M., Khan F.S., Yang M. Restormer: Efficient Transformer for High-Resolution Image Restoration. Proceedings of the 2022 IEEE/CVF Conference on Computer Vision and Pattern Recognition (CVPR).

[35] Ba J.L., Kiros J.R., Hinton G.E. (2016). Layer Normalization. arXiv.

[36] Vaswani A., Shazeer N., Parmar N., Uszkoreit J., Jones L., Gomez A.N., Kaiser L.u., Polosukhin I., Guyon I., Luxburg U.V., Bengio S., Wallach H., Fergus R., Vishwanathan S., Garnett R. (2017). Attention is All you Need. Proceedings of the Advances in Neural Information Processing Systems.

[37] Hu J., Shen L., Albanie S., Sun G., Wu E. (2020). Squeeze-and-Excitation Networks. IEEE Trans. Pattern Anal. Mach. Intell..

[38] Nair V., Hinton G.E. Rectified linear units improve restricted boltzmann machines. Proceedings of the 27th International Conference on Machine Learning (ICML-10).

[39] Hendrycks D., Gimpel K. (2023). Gaussian Error Linear Units (GELUs). arXiv.

[40] Corbetta M., Shulman G. (2002). Control of goal-directed and stimulus-driven attention in the brain. Nat. Rev. Neurosci..

[41] Hayhoe M., Ballard D. (2005). Eye movements in natural behavior. Trends Cogn. Sci..

[42] Woo S., Park J., Lee J.Y., Kweon I.S. CBAM: Convolutional Block Attention Module. Proceedings of the Computer Vision—ECCV 2018.

[43] Chen Y., Kalantidis Y., Li J., Yan S., Feng J. A^2^-Nets: Double Attention Networks. Proceedings of the 32nd Advances In Neural Information Processing Systems 31 (NIPS 2018).

[44] Gao Z., Xie J., Wang Q., Li P. Global Second-Order Pooling Convolutional Networks. Proceedings of the 2019 IEEE/CVF Conference on Computer Vision and Pattern Recognition (CVPR).

[45] Cao Y., Xu J., Lin S., Wei F., Hu H. GCNet: Non-Local Networks Meet Squeeze-Excitation Networks and Beyond. Proceedings of the 2019 IEEE/CVF International Conference on Computer Vision Workshop (ICCVW).

[46] Misra D., Nalamada T., Arasanipalai A.U., Hou Q. Rotate to Attend: Convolutional Triplet Attention Module. Proceedings of the 2021 IEEE Winter Conference on Applications of Computer Vision (WACV).

[47] Huang Z., Wang X., Wei Y., Huang L., Shi H., Liu W., Huang T.S. (2023). CCNet: Criss-Cross Attention for Semantic Segmentation. IEEE Trans. Pattern Anal. Mach. Intell..

[48] Hou Q., Zhang L., Cheng M.M., Feng J. Strip Pooling: Rethinking Spatial Pooling for Scene Parsing. Proceedings of the 2020 IEEE/CVF Conference on Computer Vision and Pattern Recognition (CVPR).

[49] Sun K., Xiao B., Liu D., Wang J. Deep High-Resolution Representation Learning for Human Pose Estimation. Proceedings of the 2019 IEEE/CVF Conference on Computer Vision and Pattern Recognition (CVPR).

[50] Pan H., Gao F., Dong J., Du Q. (2023). Multiscale Adaptive Fusion Network for Hyperspectral Image Denoising. IEEE J. Sel. Top. Appl. Earth Obs. Remote Sens..

[51] Sajjadi M.S.M., Schölkopf B., Hirsch M. EnhanceNet: Single Image Super-Resolution Through Automated Texture Synthesis. Proceedings of the 2017 IEEE International Conference on Computer Vision (ICCV).

[52] Wang Z., Bovik A., Sheikh H., Simoncelli E. (2004). Image quality assessment: From error visibility to structural similarity. IEEE Trans. Image Process..

[53] Zhao H., Gallo O., Frosio I., Kautz J. (2017). Loss Functions for Image Restoration With Neural Networks. IEEE Trans. Comput. Imaging.

[54] Starck J.L., Fadili J., Murtagh F. (2007). The Undecimated Wavelet Decomposition and its Reconstruction. IEEE Trans. Image Process..

[55] Ma K., Duanmu Z., Wu Q., Wang Z., Yong H., Li H., Zhang L. (2017). Waterloo Exploration Database: New Challenges for Image Quality Assessment Models. IEEE Trans. Image Process..

[56] Gallego A.J., Pertusa A., Gil P. (2018). Automatic Ship Classification from Optical Aerial Images with Convolutional Neural Networks. Remote Sens..

[57] Jang G., Lee W., Son S., Lee K. C2N: Practical Generative Noise Modeling for Real-World Denoising. Proceedings of the 2021 IEEE/CVF International Conference on Computer Vision (ICCV).

[58] Zhang K., Zuo W., Gu S., Zhang L. Learning Deep CNN Denoiser Prior for Image Restoration. Proceedings of the 2017 IEEE Conference on Computer Vision and Pattern Recognition (CVPR).

[59] Zhang K., Li Y., Zuo W., Zhang L., Van Gool L., Timofte R. (2022). Plug-and-Play Image Restoration With Deep Denoiser Prior. IEEE Trans. Pattern Anal. Mach. Intell..

[60] Schmidt U., Roth S. Shrinkage Fields for Effective Image Restoration. Proceedings of the 2014 IEEE Conference on Computer Vision and Pattern Recognition.

[61] Chen Y., Pock T. (2017). Trainable Nonlinear Reaction Diffusion: A Flexible Framework for Fast and Effective Image Restoration. IEEE Trans. Pattern Anal. Mach. Intell..

[62] Tian C., Xu Y., Fei L., Wang J., Wen J., Luo N. (2019). Enhanced CNN for image denoising. Caai Trans. Intell. Technol..

[63] Godard C., Mac Aodha O., Firman M., Brostow G.J. Digging into self-supervised monocular depth estimation. Proceedings of the IEEE/CVF International Conference on Computer Vision.

